# Exploring Cancer Dependency Map genes and immune subtypes in colon cancer, in which *TIGD1* contributes to colon cancer progression

**DOI:** 10.18632/aging.204859

**Published:** 2023-07-13

**Authors:** Guoyang Zhang, Zongfeng Feng, Qingwen Zeng, Ping Huang

**Affiliations:** 1 Department of General Surgery, First Affiliated Hospital of Nanchang University, Nanchang, China; 2 Medical Innovation Center, The First Affiliated Hospital of Nanchang University, Nanchang, China; 3 Department of Nutrition, The First Affiliated Hospital of Nanchang University, Nanchang, China

**Keywords:** colon cancer, cancer dependency map, CRISPR-Cas9, prognostic signature, CDM immune subtypes

## Abstract

Background: Tumour-dependent genes identified in CRISPR-Cas9 screens have been widely reported in Cancer Dependency Maps (CDMs). CDM-derived tumour-dependent genes play an important role in tumorigenesis and progression; however, they have not been investigated in colon cancer (CC).

Methods: CDM genes overexpressed in CC were identified from the TCGA-COAD dataset and CDM platform. A CDM signature and prognostic nomogram were constructed by Lasso Cox regression and multivariate Cox analyses. A weighted correlation network analysis (WGCNA) and consensus clustering were used to define coexpressed genes with CDM risk scores and to determine two new immune subtypes. A comprehensive investigation was performed between the two subtypes and immune regulation, the immune microenvironment and the impact of immunotherapy.

Results: First, 1304 overexpressed CDM genes were identified. Then, a CDM signature with five cancer-dependent genes (*MMS19, NOP14, POLRMT, SNAPC5* and *TIGD1*) and a prognostic nomogram were constructed, and they demonstrated robust predictive performance and a close relationship with clinical characteristics in different CC datasets. Patients with high CDM risk scores showed worse survival outcome and weaker response to chemotherapy. Additionally, *TIGD1* genes were oncogenes that affected the CC cell cycle, according to cell functional experiments that involved the suppression of the *TIGD1* gene. Furthermore, WGCNA and consensus clustering were used to define coexpressed genes with CDM risk scores and to determine two new immune subtypes. Finally, systematic investigations were conducted with the relationship between the CDM subtypes and immune regulation.

Conclusions: This study constructed a CDM signature consisting of five risk genes that predict survival in CC patients. In addition, the immune subtypes provided valuable insights into immunotherapy for CC patients. *TIGD1*, as an oncogene, is independent prognostic factors for CC, and contributes to CC progression.

## INTRODUCTION

Recent studies have suggested that tumorigenesis is closely related to the alteration of genetic material and the deterioration of the environment of the organism [1, 2]. Moreover, environmental degradation can cause somatic cell biological dysfunction, such as impaired DNA damage repair [3], abnormal activation or inhibition of signalling pathways [4], and dysregulation of protein production and degradation [5], which can lead to the deterioration of normal epithelial cells into tumour cells. The most common gastrointestinal cancer is colon cancer (CC), and the number of new cases is increasing every year [6]. However, the pathogenesis of CC has yet to be fully elucidated. Normal intestinal mucosal epithelium-proliferating microadenoma-early adenoma-intermediate adenoma-advanced adenoma-cancer-cancer metastasis, seems to be the growth history chain of most CC. How to interrupt this growth chain and identify oncogenes associated with tumour growth have become the keys to preventing and treating CC.

The Cancer Dependency Map (CDM) project [7], initiated by scientists at the Broad Institute and Dana-Farber Cancer Institute, aims to use RNA interference (RNAi) and CRISPR-Cas9, two gene-silencing technologies, to uncover genes that drive cancer cell proliferation. The CDM project’s dataset is updated quarterly, and data published in 2017 revealed that the screening of hundreds of cancer cell lines was completed and that 769 strongly associated tumour-dependent genes were identified [8]. Data released in April 2022 show that the project has screened 1,393 cell lines for 33 cancers, and identified 19,177 genes. Due to its excellent non-off-target effects, CRISPR-Cas9 has been employed extensively in gene editing in recent years [9]. Therefore, the basis of our study was built on CRISPR-Cas9 technology and the CDM project.

Tumour cells of different origins, and even cells of the same tumour, have different genomic mechanisms, which determines the diversity and complexity of tumour-dependent genes. This poses a great challenge to screening for tumour-dependent genes and exploring the relationship between genes. Methyl-methanesulfonate sensitivity 19 (*MMS19*) has been found in a variety of tumours [10–12] and is involved in DNA metabolism and genomic stability through the regulation of nucleotide excision repair and Fe-S protein expression, thereby affecting tumor progression [13]. Recently, studies regarding the influence of nucleolar complex protein 14 (*NOP14*) on cancers have been conducted, and this protein has been shown to play an important role in cancer migration and invasion [14, 15]. In addition, Yin et al. [16] discovered that Trigger transposable element-derived 1 (*TIGD1*) is coupled with a malignancy survival and associated with regulating cell-cycle progression in human cancer. Mitochondrial RNA polymerase (*POLRMT*) is often reported as a target protein for the treatment of malignant tumours [17], however snRNA-activating protein complex 5 (*SNAPC5*), also named *SNAP19* [18], has rarely been reported in tumours.

We constructed a valid and robust CDM signature and predictive nomogram by organically combining RNA sequencing data from The Cancer Genome Atlas–colon adenocarcinoma (TCGA-COAD) tumour samples with data on the tumour-dependent gene interference efficiency of CC cells in the CDM; the signature and nomogram were validated with both internal and external data. Furthermore, two novel immune subtypes were established by weighted correlation network analysis (WGCNA) and consensus clustering, that can be used to precisely determine whether patients will benefit from immunotherapy. Finally, functional experiments revealed thatTIGD1 genes was oncogenes that impacted the CC cell cycle.

## MATERIALS AND METHODS

### Identifying CDM genes from COAD cells

Interference efficiency files identified by CRISPR-Cas9 for 55 CC cell lines with dependent genes were downloaded from the CDM (https://depmap.org/portal/) [19]. As of April 2022, the CDM has validated 19,177 genes covering 1,393 cell lines from 33 malignancies, making it the most comprehensive research database of tumour cells available. A negative value of interference efficiency indicated that knockdown of a gene inhibited the growth of tumour cells, while a positive value promoted cell growth. When the interference efficiency of a gene was less than -0.5, knockdown of the gene was considered to significantly inhibit the growth of tumour cells, and it was referred to as a cancer dependent gene. A total of 1,613 genes were considered CC dependent genes, as their interference efficiency averaged less than -0.5 in 55 types of CC cells.

### Downloading and preprocessing of TCGA and GEO data

TCGA-COAD RNA-Seq and clinical information data, including 452 samples, were downloaded from TCGA (https://portal.gdc.cancer.gov) in March 2022. The format of RNA-Seq expression data was converted from FPKM to TPM, which is more in consistent with the GEO (Gene Expression Omnibus) (https://www.ncbi.nlm.nih.gov/geo/) chip data and provides more convenience for joint analysis of TCGA and GEO data. The GSE17536 microarray dataset contained 177 CC sample RNA-Seq expression and clinical data (Supplementary Table 1). Subsequently, CC datasets from different data sources were log2 (value+1) normalized using the “limma” and “sva” bioconductor packages in the 4.10 R software.

### Intersecting genes among the upregulated and CDM genes

The “limma” package was used to compare the differences in gene expression between normal tissue and tumour tissue for the corrected TCGA-COAD data and a p value less than 0.05 and log2-fold change (logFC) greater than 1 indicated upregulated COAD genes. The “VennDiagram” package was used to identify 1,034 intersecting genes among the upregulated and CDM genes.

### Functional enrichment analysis of intersecting genes

Kyoto Encyclopedia of Genes and Genomes (KEGG) and Gene Ontology (GO) analyses were performed using the “clusterProfiler”, “org.Hs.eg.db”, “enrichplot” and “ggplot2” packages.

### Constructing and validating the CDM signature and nomogram

First, univariate Cox hazard analysis was used to screen for prognostic genes via the “limma” and “survival” packages. Then, the TCGA-COAD data were randomly separated into a training group and a test group at a 1:1 ratio. Next, Lasso Cox regression analysis was used to reduce the effect of multicollinearity among multiple genetic variables. Multivariate Cox analysis was performed to construct the CDM risk model based on the genes cohort, TCGA-COAD all cohort and GSE17536 cohort was evaluated using a time-dependent receiver operating characteristic (ROC) curve with the R package “timeROC”. Based on the signature, the risk score of each sample in the TCGA-COAD and GSE17536 cohorts was determined, and risk curves and patient survival status corresponding to each sample were plotted along with the expression heatmap of the five genes in the signature. Subsequently, the Kaplan–Meier plot was used to investigate the overall survival heterogeneity between the high- and low- risk groups. Finally, a nomogram evaluating the survival time of CC patients was created and validated by R package “rms”, combined with patient clinical characteristics. Decision curve analysis (DCA) and ROC curves were calculated to detect the accuracy of the nomogram using the R packages “ggDCA” and “timeROC”.

### Signature gene interference effects from CRISPR-Cas9 screens

The interference efficiency of each gene in CC cells was collated from the CDM, and the interference efficiency of the *SNAPC5, TIGD1, NOP14, MMS19* and *POLRMT* genes in the signature was extracted and visualized using GraphPad 7.0 software. We also further calculated the efficiency of the risk model in determining cell growth interference in common CC cells, including DLD1, HCT116, HT29 and SW620 cells.

### Immunohistochemical (IHC), immunofluorescence (IF) and Western blotting (WB)

The Human Protein Atlas (HPA) [20] (https://www.proteinatlas.org/) has been developing of antibodies to human-encoded proteins, and the database includes a large number of immunohistochemical sections of normal and pathological human tissues and partial immunofluorescence sections of cells. The protein expression and intracellular localization of five genes were exhibited on the HPA platform. Western blotting was performed according to a previous study [21]. The following antibodies were used in this study: GAPDH (No. 60004-1-Ig, 1:10000 dilution, Proteintech, China), MMS19 (No: 66049-1-Ig, 1:1000 dilution, Proteintech, China), NOP14 (No:26854-1-AP, 1:500 dilution, Proteintech, China), POLRMT (No: A15605, 1:500 dilution, ABclonal, China), SNAPC5 (No:17272-1-AP, 1:500 dilution, Proteintech, China), and TIGD1 (No:13833-1-AP, 1:500 dilution, Proteintech, China).

### Cell culture, infection, siRNAs, and cell function assays

The COAD cells DLD1, HT29, HCT116, and SW620 and the normal-derived colon mucosal cells (NCM460) were maintained in Dulbecco’s modified Eagle’s medium (DMEM) with 10% foetal bovine serum (HyClone GE Healthcare Life Sciences, Logan, UT, USA) at 37° C in a 5% CO_2_ atmosphere. Small interfering RNA (siRNA) targeting the TIGD1 genes and a negative control were purchased from GenePharma Company (Shanghai, China). These siRNAs and negative control RNA were transfected into HCT116 and SW620 cells. The sequences of siRNAs that disturbed *TIGD1* gene are shown in Table 1.

**Table 1:** *TIGD1* gene siRNA sequence.

**Gene name**	**Sequences**	
	**Sense (5'-3')**	**Antisense (5'-3')**
TIGD1-988	CCAGAACAAAGCCCUAACUTT	AGUUAGGGCUUUGUUCUGGTT
TIGD1-1170	CAGAAGCUUUAGCUAAGAUTT	AUCUUAGCUAAAGCUUCUGTT
Negative control	UUCUCCGAACGUGUCACGUTT	ACGUGACACGUUCGGAGAATT

Colony formation: The experimental procedure can be found in our previous study [21]. Cell proliferation assay: A total of 1000 cells were inoculated in 96-well culture plates, and 10 μl Cell Counting Kit (CCK8) reagent (Beyotime Biotechnology Company, Shanghai, China) was added to each well at hours 24, 48, 72, and 96. The cells were cultured for 2 hours, the absorbance was detected at 450 nm, and the growth curve was plotted. Cell cycle assay: The transfected cells were inoculated into 6-well plates, and when the cell abundance reached 80%, the cells were digested and fixed in 70% alcohol overnight. The next day, the fixed cells were stained with a cell cycle kit (Dojindo Laboratories, Japan) and the cell cycle was detected by flow cytometry.

### Drug IC50 analysis based on the CDM signature

The half maximal inhibitory concentration (IC50) values for dozens of drugs were evaluated in both the high- and low- risk groups with the R package “pRRophetic” [22].

### Screening hub oncogene in CDM risk genes

Sangerbox 3.0 (http://www.sangerbox.com/) [23] was used to analysis *TIGD1* expression, the relationship with clinical characteristic and overall survival in TCGA-COAD dataset. The PrognoScan (http://www.prognoscan.org/) [24] database was used to analyse the prognostic value of gene from the GSE17536 dataset.

### Weighted gene coexpression network analysis (WGCNA) and CDM immune subtypes construction

WGCNA is a method for clustering genes with similar expression patterns and further investigating the association between the modules of various clusters and clinical phenotypes, and is therefore widely used in the analysis of the correlation between clinical phenotypes and gene expression in tumours. WGCNA, as an algorithm for gene coexpression networks, involves three main principles: 1) assuming that gene expression obeys a scale-free distribution, 2) defining the expression matrix of genes and the adjacency function formed by the gene network, and 3) calculating the dissimilarity coefficients of different nodes to construct hierarchical clustering trees. Following the risk scoring of the TCGA-COAD samples, the risk score was incorporated into the WGCNA as a significant clinical phenotype with the following relevant parameters: minModuleSize = 30, MEDissThres = 0.3 and R package “WGCNA”. After obtaining the black module genes associated with the risk model, we acquired the differentially expressed black module genes by intersecting the black module genes with the differentially expressed genes, and then divided the TCGA samples into two types by the R package “ConsensusClusterPlus”. The two types were termed CDM subtypes.

### Immunity analysis of CDM-associated immune subtypes

TCGA-COAD expression data were used to investigate the relationship between the two CDM-associated subtypes and immunity. First, we calculated the immune microenvironment score of the samples by the R package “estimate”; second, we used the Microenvironment Cell Populations counter (MCPcounter) algorithm to calculate the immune cell score of the samples by the R package “MCPcounter” [25]; finally, we estimated the difference between subtypes by the R package “limma” and drew a violin map. Based on the strong association between CDM subtypes and immune cell infiltration, we downloaded TCGA-COAD immunotherapy data from The Cancer Imaging Archive [26] (TCIA: https://tcia.at/home) and performed subtype correlation analysis with programmed cell death protein 1 (PD1) and cytotoxic T-lymphocyte-associated protein 4 (CTLA4) immunotherapy using the R package “ggpubr”. Sankey diagrams were used to explore the association between the CDM subtypes and immune subtypes commonly found in clinical tumours: namely, wound healing (C1), IFN-dominant (C2), inflammatory (C3), lymphocyte depleted (C4), immunologically quiet (C5) and TGF-dominant (C6), by the R packages “ggplot2”, “ggalluvial” and “dplyr”.

### Data availability statement

The inquiries of original data can be directed to the corresponding authors.

### Code availability

Bioinformatics analysis was based on R 4.1.0, and the involved R packages were described in detail in the methods section. All codes were written and reviewed by GY.Z and ZF.F, respectively. GY.Z and ZF.F had the ultimate power of interpretation and ownership of the code.

### Consent for publication

All authors agreed to be published.

## RESULTS

### CDM analysis with CC flow chart

**Figure 1:**
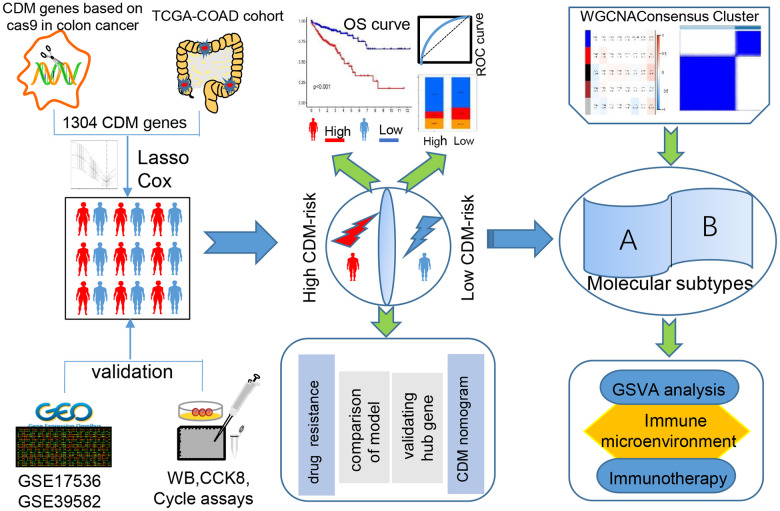
Cancer Dependency Map (CDM) with colon cancer (CC) analysis flow chart.

A series of procedures was designed to investigate the prognosis of CC based on the characteristics of the CDM genes (Figure 1).

### Expression of upregulated CDM genes and functional enrichment analysis

After normalization of the TCGA-COAD data, 8,179 genes with upregulated expression in CC were screened by the selection criteria of p<0.05 and logFC>1. Additionally, 1,613 CC-dependent genes were discovered by collating interference efficiency with in 55 type of CC cells. A total of 1,304 CDM genes were differentially expressed in CC (Supplementary Figure 1A), and the heatmap in Supplementary Figure 2 depicted their differential expression in colon normal tissue and CC tissue. To explore the signalling pathways and functional analysis of the different CDM genes in CC in depth, we performed KEGG and GO analyses. As shown in Supplementary Figure 1B, five significant pathways cell cycle, nucleocytoplasmic transport, ribosome, ribosome biogenesis in eukaryotes and spliceosome were enriched. Biological process analysis revealed that different CDM genes were involved in ncRNA metabolic processes, ncRNA processing ribonucleoprotein complex biogenesis and ribosome biogenesis (Supplementary Figure 1C).

### Construction and validation of the CDM signature

First, 446 TCGA-COAD samples, excluding eight samples with missing clinical data, were randomly assigned to the training and testing cohorts in a 1:1 ratio, with 224 cases in the training cohort and 222 cases in the testing cohort. Next, univariate Cox hazard analysis was used to regress the differentially expressed CDM genes in the TCGA-COAD data according to a p<0.05 criterion, and 29 genes were discovered to be substantially associated with the survival prognosis of the TCGA-COAD patients (Supplementary Table 1). Twenty-nine genes are not conducive to medical detection and promotion. As a result, Lasso Cox regression analysis was performed to filter for precise and valid prognosis-related genes, and the confidence interval under each lambda is shown in Supplementary Figure 3A. The prognosis-related genes were narrowed down to seven genes (Supplementary Figure 3B). Multivariate Cox analysis was employed to model the prognostic assessment of the five CDM genes. The five CDM-gene model formula was as follows: CDM risk score = 0.0538052 × exp*^SNAPC5^* + 0.925782 × exp*^TIGD1^* + -1.54469 × exp*^NOP14^* + 1.559607 × exp*^MMS19^* + 0.592523 × exp*^POLRMT^*.

**Figure 2:**
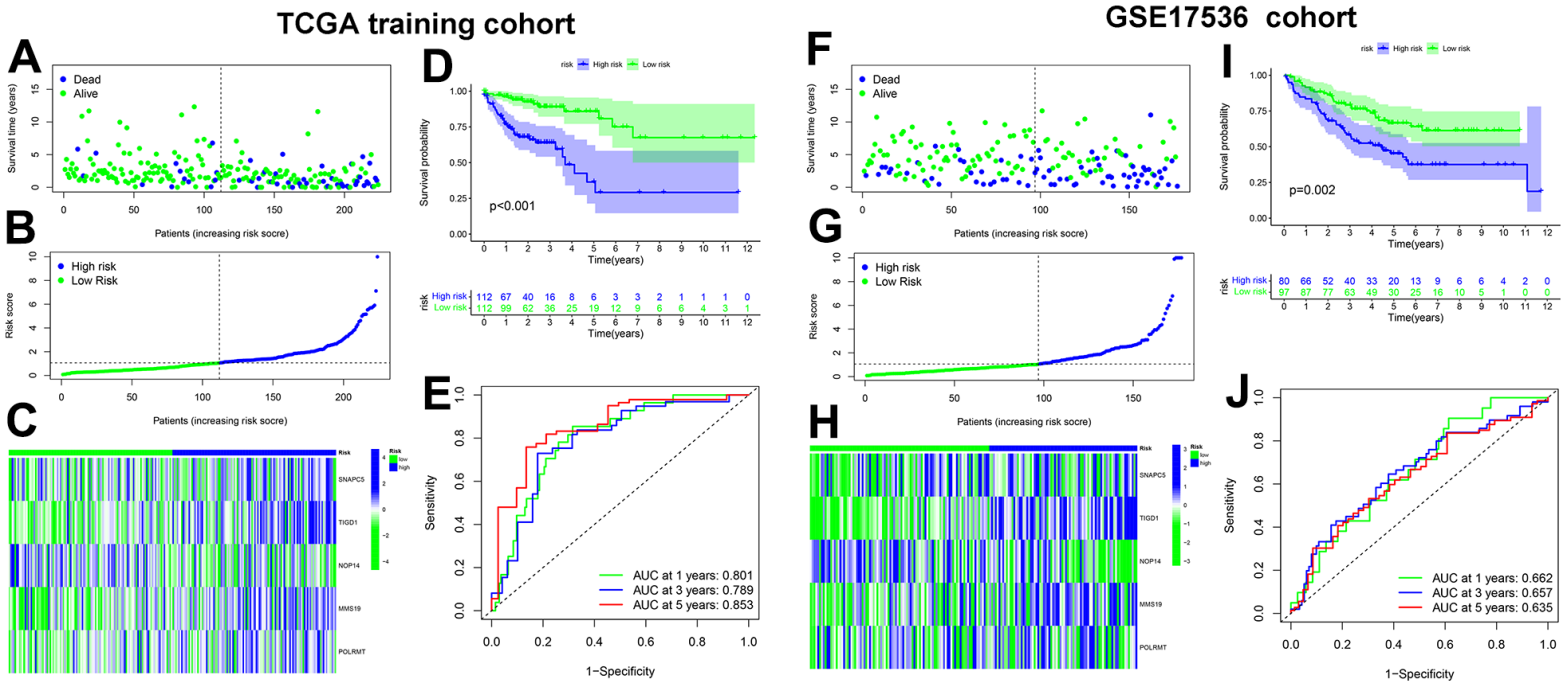
**Cancer Dependency Maps (CDMs) signature construction and validation in TCGA training cohort and GSE17536 cohort.** (**A**) Survival scatter plot in TCGA training cohort. A dot represents a CC patient (blue represents Dead, Green represents alive). Dotted lines show the median of risk score that dichotomize patients into high and low groups. (**B**) Risk score plot in TCGA training cohort. The patients of CC are ordered by the risk score of the CDM signature. Dotted lines show the median of risk score that dichotomize patients into high (blue) and low (green) groups. (**C**) Heatmap of five CDM signature genes (*SNAPC5*, *TIGD1*, *NOP14*, *MMS19* and *POLRMT*) in TCGA training cohort. Blue represents high-CDM risk group and green represents low-CDM risk group. (**D**) Kaplan-Meier plot in TCGA training cohort (*p*<0.001). Survival curves for high (blue) and low (green) risk-CDM groups dichotomized at the median of risk-CDM score are plotted. 95% confidence intervals for each group are also indicated by shadow area. (**E**) The ROC curve of CDM signature in TCGA training cohort. Green show one year AUC value (AUC value=0.801), blue show three years AUC value (AUC value=0.789) and red show five years AUC value (AUC value=0.853). (**F**) Survival scatter plot in GSE17536 cohort. (**G**) Risk score plot in GSE17536 cohort. (**H**) Heatmap of five CDM signature genes (*SNAPC5*, *TIGD1*, *NOP14*, *MMS19* and *POLRMT*) in GSE17536 cohort. (**I**) Kaplan-Meier plot in GSE17536 cohort (*p*=0.002). (**J**) The ROC curve of CDM signature in GSE17536 cohort. One year AUC value (AUC value=0.662), three years AUC value (AUC value=0.657) and five years AUC value (AUC value=0.635).

We calculated the risk scores for the training cohort samples based on the expression level of the five CDM genes, and then determined the patient survival status distribution according to the risk scores. It was discovered that the high-risk CC population had shorter survival times in the training cohort than the low-risk group, and the AUC predictive values in the time-dependent ROC curves for 1, 3, and five years were 0.801, 0.789, and 0.853, respectively, indicating that the CDM risk signature has good survival predictive power for CC. Subsequently, the heatmap revealed that SNAPC5, TIGD1, MMS19 and POLRMT were highly expressed in the high-risk group, while NOP14 was expressed at low levels in the low-risk group (Figure 2A–2E). To determine the robustness of CDM signature, we used the same coefficients obtained from the training cohort in the internal testing cohort, all TCGA-COAD cohorts, and GSE17536 external validation cohort. The risk score evaluation, patient survival status distribution, heatmap of the five CDM genes, K-M survival curve and time-dependent ROC curves with GSE17536 external validation cohort are shown in Figure 2F–2J. Supplementary Figure 4A–4J correspond to the all TCGA-COAD cohort and partial testing TCGA-COAD cohort, respectively. The above findings showed that the CDM signature is robust, and play a consistent prognostic predictive role in various CC datasets.

### Good predictive ability of the CDM signature compared with other models

Based on a literature review, four prognostic signatures were chosen for comparison with the CDM signature, namely, the Li [27], Nie [28], Ren [29], and Xu [30] signature, were chosen for comparison with the CDM signature to TCGA-COAD cohort. To make the results more informative, we calculated risk scores for each TCGA-COAD sample based on the risk genes modelled for each individual, divided the samples into high and low risk groups, and performed survival analysis and ROC curve validation.

**Figure 3:**
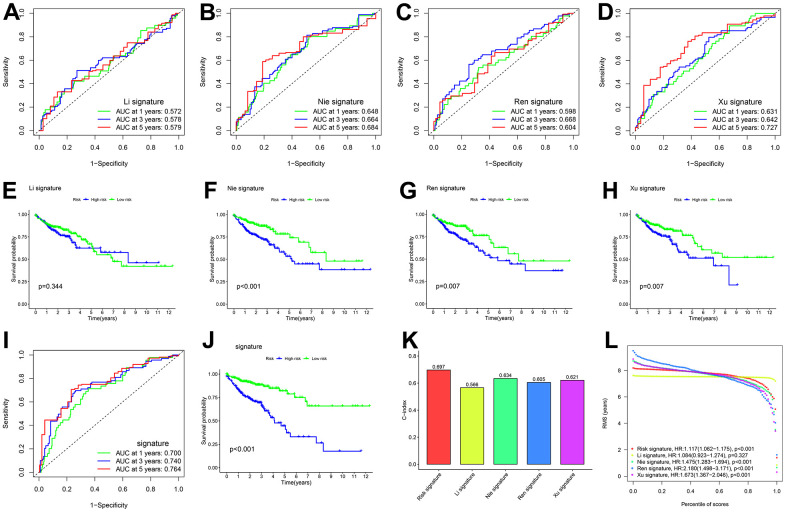
**Comparison of five CDM genes model with other models.** (**A**, **B**) The ROC curve of signatures in TCGA-COAD cohort. (**A**) Li signature (ten genes). Green show one year AUC value (AUC value=0.572), blue show three years AUC value (AUC value=0.578) and red show five years AUC value (AUC value=0.579). (**B**) Nie signature (five genes). Green show one year AUC value (AUC value=0.648), blue show three years AUC value (AUC value=0.664) and red show five years AUC value (AUC value=0.684). (**C**) Ren signature (eight genes). Green show one year AUC value (AUC value=0.598), blue show three years AUC value (AUC value=0.668) and red show five years AUC value (AUC value=0.604). (**D**) Xu signature (eight gene). Green show one year AUC value (AUC value=0.631), blue show three years AUC value (AUC value=0.642) and red show five years AUC value (AUC value=0.727). (**E**–**H**) Kaplan-Meier plot in all TCGA –COAD cohort (p<0.001). Survival curves for high (blue) and low (green) risk groups dichotomized at the median of risk score are plotted. (**E**) shows Li signature (p=0.344), (**F**) shows Nie signature (p<0.001), (**G**) shows Ren signature (p=0.007) and (**H**) shows Xu signature (p=0.007). (**I**) The ROC curve of CDM signature in all TCGA-COAD cohort. Green show one year AUC value (AUC value=0.700), blue show three years AUC value (AUC value=0.740) and red show five years AUC value (AUC value=0.764). (**J**) Kaplan-Meier plot in all TCGA –COAD cohort (p<0.001). Survival curves for high (blue) and low (green) risk-CDM groups dichotomized at the median of risk-CDM score are plotted. (**K**) C-index of the five prognostic risk models. The higher the C-index, and the more reliable the signature. Red shows CDM risk signature (C-index=0.697). Yellow shows Li signature (C-index=0.566). Cyan shows Nie signature (C-index=0.634). Blue shows Ren signature (C-index=0.605), Dull red shows Xu signature (C-index=0.621). (**L**) The RMS analysis of the five prognostic risk signature. Red shows CDM risk signature. Yellow shows Li signature. Cyan shows Nie signature. Blue shows Ren signature, Dull red shows Xu signature.

When comparing Figure 3A–3D, 3I, the AUC of the 1-year, 3-year, and 5-year time-dependent ROC curves for the CDM risk models was clearly higher than that of the other four signature. Similarly, the survival curves of the CDM signature outperformed those of the others as shown in Figure 3E–3H, 3J. The consistency index (c-index) and RMS values were introduced to further compare the prognostic prediction performance of these models for TCGA-COAD cohort. As shown in Figure 3K, 3L, the CDM signature seemed to have the highest c-index of all of the models, and the RMS values were on par with them. A series of verifications and comparisons have proven that the performance of the CDM signature overall was better than that of the other four. Furthermore, and perhaps most importantly, the CDM signature employs fewer genes to produce more accurate prediction results with good tractability for clinical replication.

### Clinical correlation analysis and the nomogram improves CDM signature predictive ability in CC

**Figure 4:**
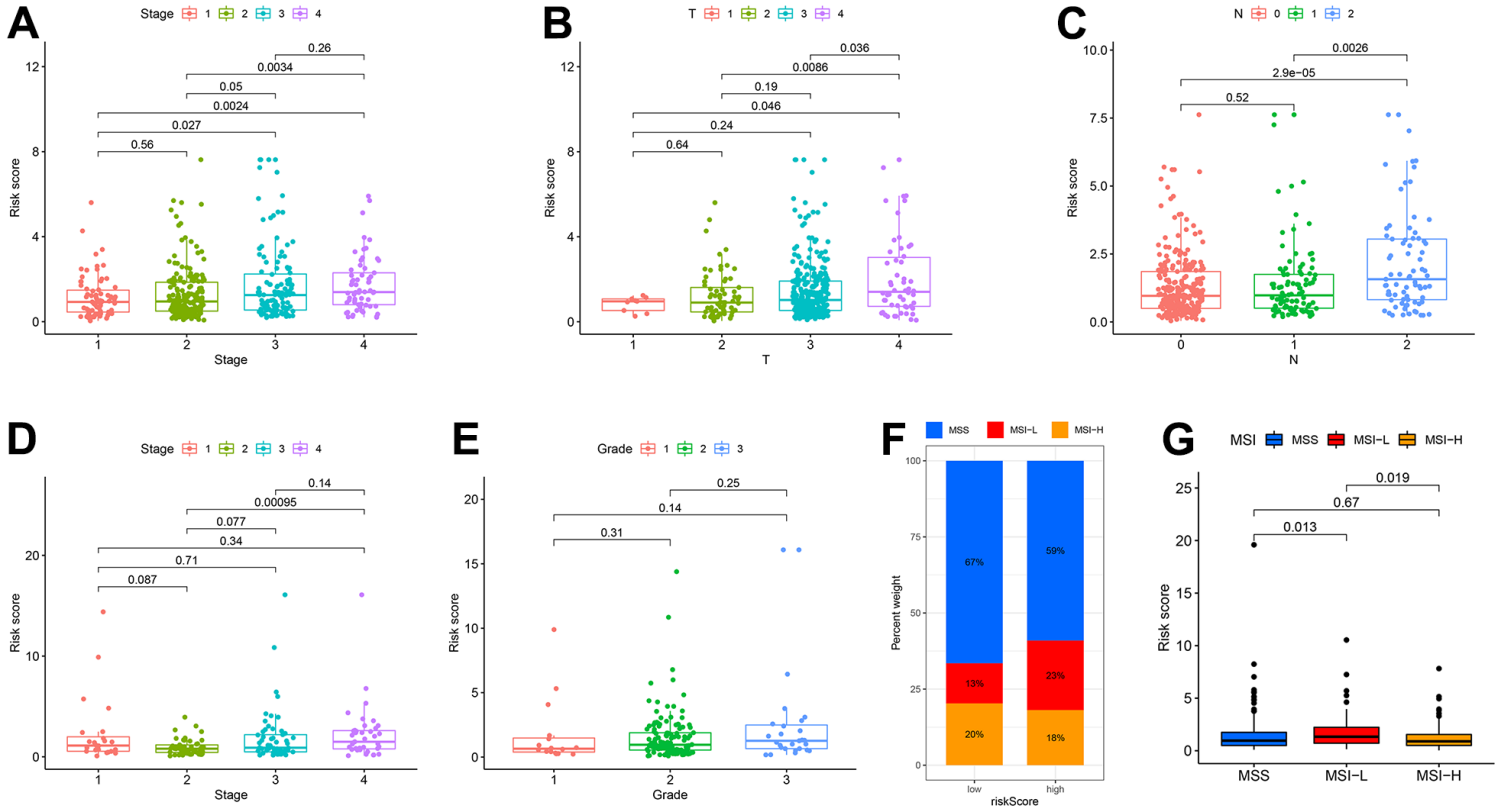
**Correlation among clinicopathological classifications and CDM signature risk scores.** (**A**) Boxplot. Correlation between tumour stage and risk-CDM score in TCGA-COAD cohort. Pink represents I stage, green represents II stage, Cyan represents III stage and violet represents IV stage. (**B**) Boxplot. Correlation between depth of tumor invasion (T) and risk-CDM score in TCGA-COAD cohort. Pink represents T1, green represents T2, cyan represents T3 and violet represents T4. (**C**) Boxplot. Correlation between lymph node metastasis (N) and risk-CDM score in TCGA-COAD cohort. Pink represents N0 (not metastasis), green represents N1 (1-3 regional lymph node metastases) and blue represents N2 (4 or more regional lymph node metastases). (**D**) Boxplot. Correlation between tumour stage and risk-CDM score in GSE17536 cohort. Pink represents I stage, green represents II stage, Cyan represents III stage and violet represents IV stage. (**E**) Boxplot. Correlation between grade and risk-CDM score in GSE17536 cohort. Pink represents G1 (good), green represents G2 (moderate) and blue represents G3 (poor). (**F**) Microsatellite stability histogram. MSS (blue), MSI-L (red) and MSI-H (orange) of High/Low risk group samples in TCGA-COAD cohort. (**G**) Boxplot. Correlation between microsatellite stability and risk-CDM score in TCGA-COAD samples. MSS (blue), MSI-L (red) and MSI-H (orange).

To illustrate the clinical utility of the CDM signature more fully and effectively, we explored the association between the signature and clinical features, and further constructed and evaluated nomogram by integrating the signature with clinical features. In terms of age and gender, based on an analysis of the CDM genes in the TCGA-COAD and GSE17536 datasets, there was no significant difference between the high- and low- risk groups (Supplementary Figure 3E–3H). However, we discovered that the worse the tumour stage was, the higher the patient CDM risk scores (Figure 4A, 4D). According to the TCGA-COAD dataset, the depth of tumour infiltration (T) and the number of lymph node metastases (N) were related to the CMD risk scores (Figure 4B, 4C). However, there was no significant difference between the CDM risk scores and tumour grades based on the GES17536 dataset (Figure 4E). Microsatellite instability (MSI) is a clinically significant marker that results from a functional defect in DNA mismatch repair in tumour tissue for CC. There is substantial evidence that microsatellite instability high (MSI-H) is a prognostic marker in patients with stage II colorectal cancer [31, 32], but there is also evidence that MSI-H patients do not benefit from adjuvant chemotherapy with 5-fluorouracil (5-Fu), and MSI-H can be used as a predictive marker of the ineffectiveness of adjuvant 5-FU treatment for CC [33]. Microsatellite stability (MSS) patients accounted for 67 percent of the low-risk CDM group, microsatellite instability low (MSI-L) for 13 percent, and MSI-H for 20 percent; the composition ratios of the three in the high-risk CDM group were significantly different from those in the low-risk group, with MSS patients accounting for 59 percent, MSI-L for 23 percent, and MSI-H for 18 percent (Figure 4F). Figure 4G also shows that MSI-L patients had the highest CDM risk score, followed by MSI-H patients, and finally MSS patients, with a statistically significant difference.

**Figure 5:**
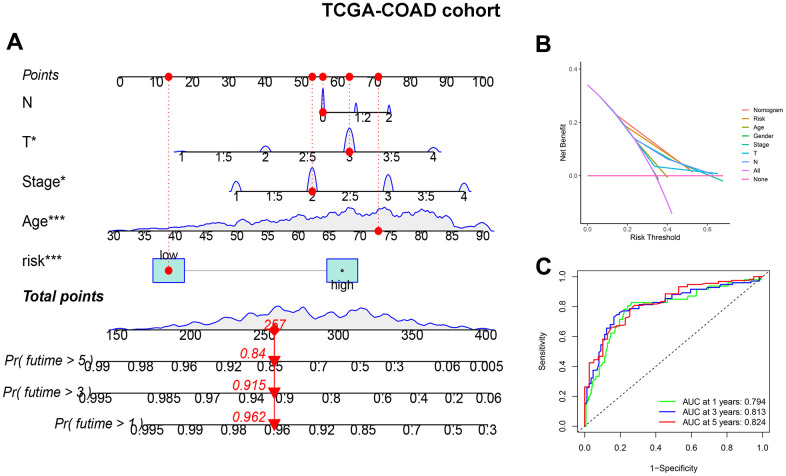
**CDM-associated nomogram construction in TCGA-COAD cohort.** (**A**) CDM-associated Nomogram for predicting 1-, 3-, and 5-year OS of patients with TCGA-COAD cohort. (**B**) The decision curve (DCA) analysis for CDM-associated nomogram in TCGA-COAD cohort. (**C**) The ROC curve of CDM-associated Nomogram in all TCGA-COAD cohort. Green show one year AUC value (AUC value=0.794), blue show three s AUC value (AUC value=0.813) and red show five years AUC value (AUC value=0.824).

To improve the accuracy of CDM signature prediction, the nomogram was constructed by combining clinical features from two datasets, TCGA-COAD and GSE17536. The nomogram in Figure 5A is more intuitive and convenient for predicting patients’ clinical prognoses, with a high operability. In addition, because the calibration plot is close to the 45° line, the column line plot in Supplementary Figure 3C, 3D performed well. Similarly, the results of the DCA considered the CDM signature and nomogram to be highly credible (Figure 5B). When compared to the AUC value of the CDM signature, the AUC of the nomogram was significantly improved (Figure 5C). Supplementary Figure 5A–5C exhibited the result of nomogram, DCA and ROC in GSE17536 cohort.

### CDM risk score improves the prediction of targeted therapy resistance

**Figure 6:**
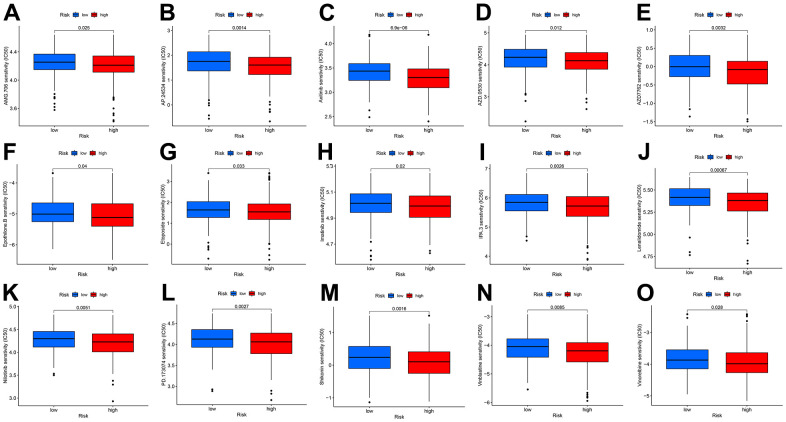
**Drug IC50 analysis in CDM risk signature (blue represents low CDM risk group, red represents high group).** (**A**) AMG.706 (*p*=0.025); (**B**) AP.24534 (*p*=0.0014); (**C**) Axitinib (*p*=6.9e^^-6^); (**D**) AZD.0530 (*p*=0.012); (**E**) AZD7762 (*p*=0.0032); (**F**) Epothilone (*p*=0.04); (**G**) Etoposide (*p*=0.033); (**H**) Imatinib (*p*=0.02); (**I**) IPA.3 (*p*=0.0026); (**J**) Lenalidomide (*p*=0.00067); (**K**) Nilotinib (*p*=0.0051); (**L**) PD.173074 (*p*=0.0027); (**M**) Shikonin (*p*=0.0016); (**N**) Vinblastine (*p*=0.0085); (**O**) Vinorelbine (*p*=0.028).

To make the CDM signature more useful for guiding the treatment of CC patients, we comprehensively explored drug resistance relationships based on the model and found that many drugs, such as molecular targeting inhibitors, immunosuppressive drugs and even some Chinese herbal medicines, had significantly different IC50 values in both the high- and low- risk groups. The drugs shown in Figure 6A–6O had higher IC50 values in the low-risk group than in the high-risk group, implying that these drugs may benefit low-risk patients to some extent.

### Expression of CDM risk genes in CC tissues and cells

**Figure 7:**
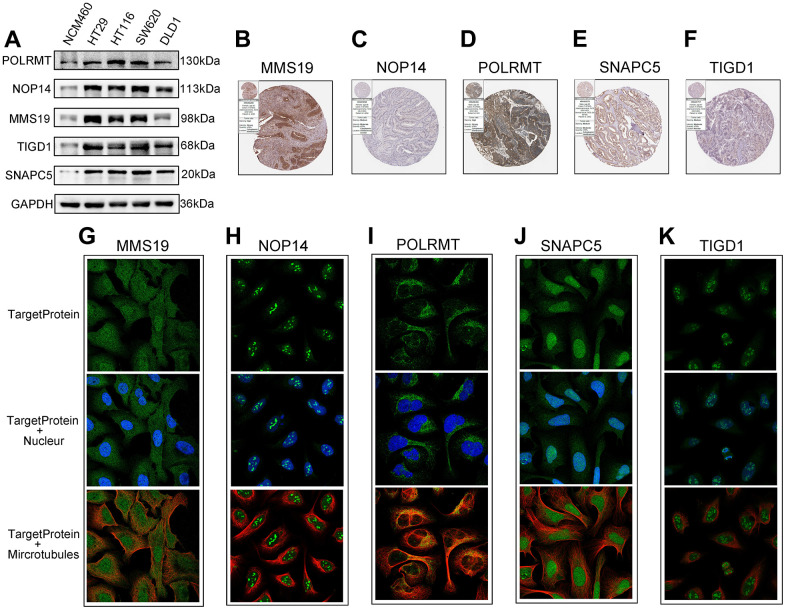
**The expression of the identified five CDM genes from the human protein atlas database (HPA).** (**A**) Western blotting. The protein expression of five CDM genes in the normal derived colon mucosal (NCM460) and colon cancer cells (HT29, HCT116, SW620 and DLD1). (**B**–**F**) Protein expressions of five CDM genes in colon cancer tissue specimens by immunohistochemical; (**B**) for MMS19; (**C**) for NOP14; (**D**) for POLRMT; (**E**) for SNAPC5; (**F**) for TIGD1. (**G**–**K**) Protein expressions and localization of five CDM genes in U2-OC cell by immunofluorescence (green represents five CDM-associated protein, blue represents nucleus and red represents microtubule); (**G**) for MMS19; (**H**) for NOP14; (**I**) for POLRMT; (**J**) for SNAPC5; (**K**) for TIGD1.

To determine the protein expression characteristics of these CDM signature genes, we investigated IHC of CC tissues, IF with U2-OC cell images from the HPA database and WB of CC cells. The WB results shown in Figure 7A, demonstrated that the protein expression of MMS19, NOP14, POLRMT, SNAPC5 and TIGD1 in NCM460 cells was far lower than that in CC cells. Additionally, the IHC results demonstrated that MMS19, NOP14, POLRMT, SNAPC5 and TIGD1 were moderately to strongly expressed in CC tissues (Figure 7B–7F). Cellular immunofluorescence localization revealed that NOP14 and TIGD1 were mainly localized in the nucleus, POLRMT was mostly found on microtubules in the cytoplasm, and MMS19 and SNAPC5 were expressed in both the nucleus and cytoplasm (Figure 7G–7K).

### Gene effect scores based on CRISPR-Cas9 and silencing TIGD1 genes inhibited CC cell proliferation

**Figure 8:**
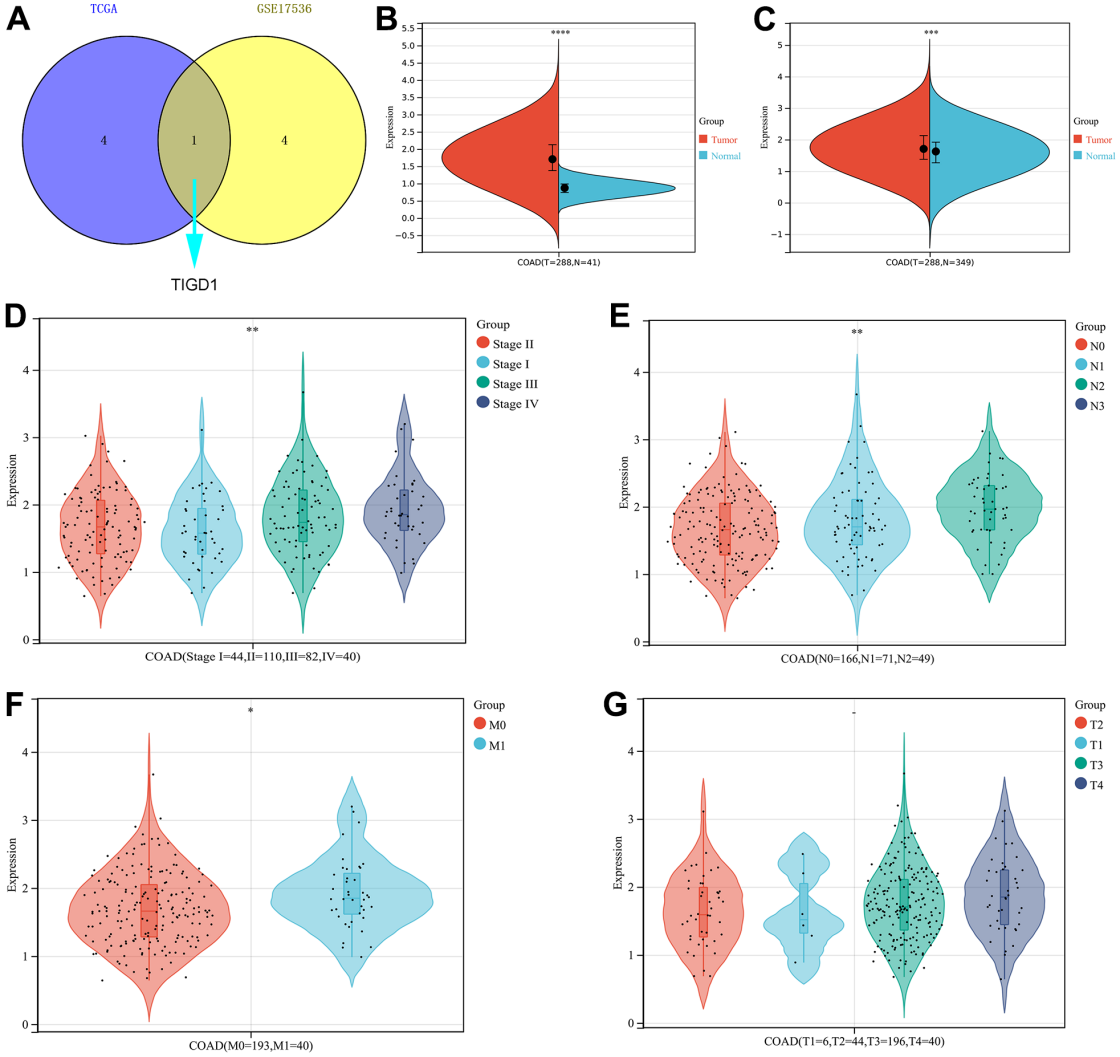
**The expression and clinicopathological classifications of *TIGD1* in TCGA-COAD cohort.** (**A**) Venn plot. Kaplan-Meier survival analysis for five CDM-associated genes in in TCGA–COAD and GSE17536 cohort. Blue represents TCGA-COAD and yellow represents GSE17536 cohort. (**B**, **C**) Violin plot. The mRNA expression of TIGD1. Red represents tumour and blue represents normal. (**B**) for TCGA-COAD dataset (p<0.0001). (**C**) for TCGA-COAD and GTEx dataset (p<0.001). (**D**) Violin plot. Correlation between tumour stage and TIGD1 expression in TCGA-COAD cohort. Pink represents I stage, green represents II stage, Cyan represents III stage and violet represents IV stage (*p*<0.01). (**E**) Violin plot. Correlation between lymph node metastasis (N) and TIGD1 expression in TCGA-COAD cohort. Pink represents N0 (not metastasis), blue represents N1 (1-3 regional lymph node metastases) and cyan represents N2 (4 or more regional lymph node metastases) (*p*<0.01). (**F**) Violin plot. Correlation between metastasis (M) and TIGD1 expression in TCGA-COAD cohort. Pink represents M0 (not metastasis), blue represents M1 (metastases) (*p*<0.05). (**G**) Violin plot. Correlation between depth of tumor invasion (T) and TIGD1 expression in TCGA-COAD cohort. Pink represents T1, blue represents T2, Cyan represents T3 and violet represents T4 (*p*>0.05).

**Figure 9:**
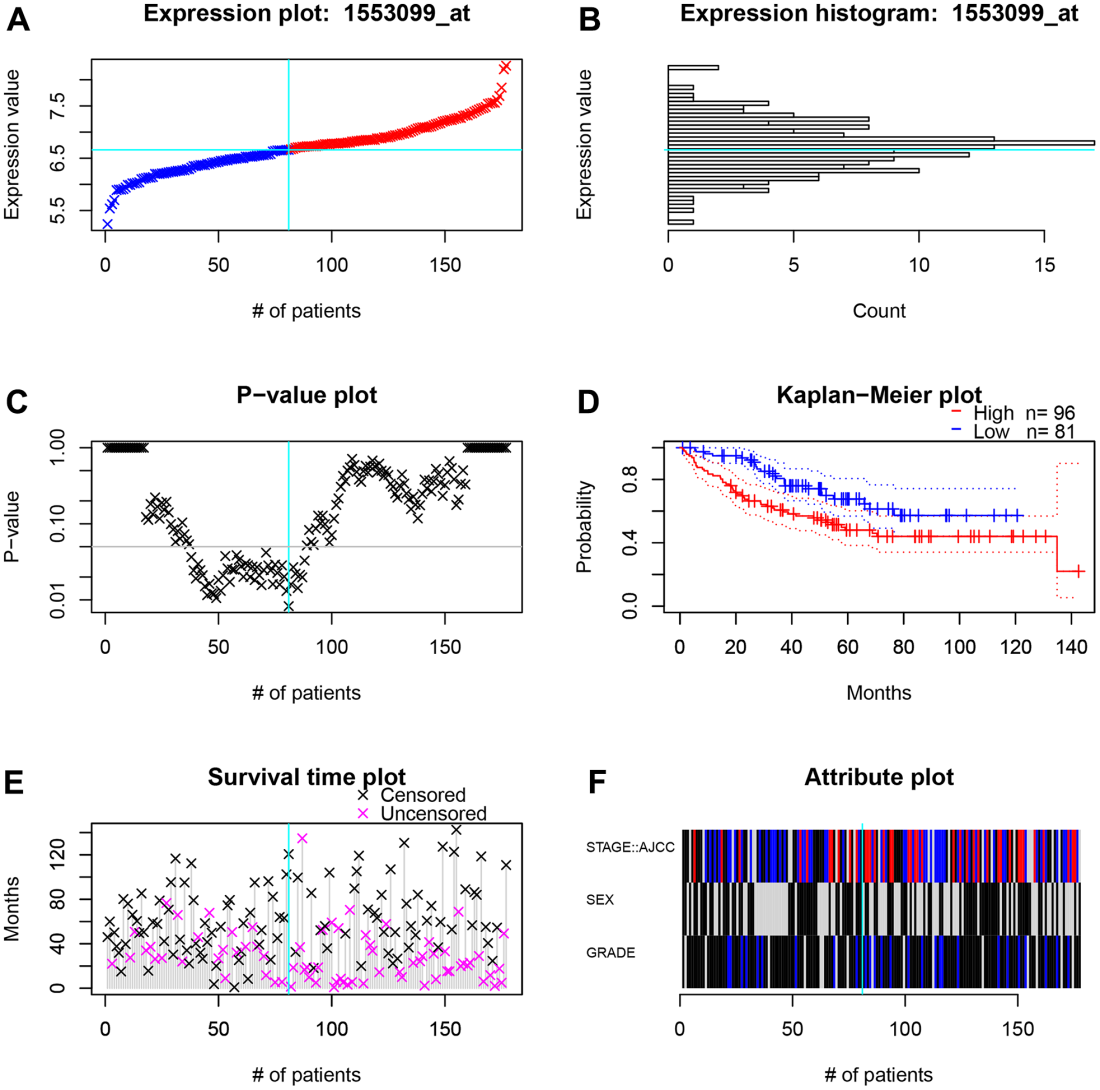
**The survival and clinicopathological classifications of *TIGD1* in GSE17536 cohort.** (**A**) Expression plot. CC patients are ordered by the TIGD1 expression. Cyan lines show the optimal cutpoints that dichotomize patients into high (red) and low (blue) expression groups. (**B**) TIGD1 expression histogram. The line of the optimal cutpoint is also shown (cyan). (**C**) P-value plot of *TIGD1*. For each potential cutpoint of expression measurement, patients are dichotomized and survival difference between high and low expression groups is calculated by log-rank test. The cutpoint to minimize the P-value is determined and indicated by the cyan line. (**D**) Kaplan-Meier plot of TIGD1. Survival curves for high (red) and low (blue) expression groups dichotomized at the optimal cutpoint are plotted. 95% confidence intervals for each group are also indicated by dotted lines. (**E**) Survival scatter plot of TIGD1. A dot represent a CC patient (pink represents uncensored, black represents censored). (**F**) Heatmap of *TIGD1* expression among stage, sex and grader. Black represents low expression and red represents high expression.

To further screen for oncogenes, the survival performance of five risk genes was analyzed in both TCGA-COAD and GSE17536 datasets (Supplementary Figure 6A–6J). Taking into account the expression differences, only *TIGD1* has excellent predictive power in both datasets (Figure 8A–8C). The clinicopathological characteristics and the expression of TIGD1 are closely correlated. For instance, the higher the expression of TIGD1, the more advanced the tumour stage and the more likely to lymph node metastasis and distant metastasis; while, tumour infiltration depth not significant (Figure 8D–8G). Subsequently, the prognostic value of *TIGD1* gene was further verified by the ProgScan in the GSE17536 dataset (Figure 9).

**Figure 10:**
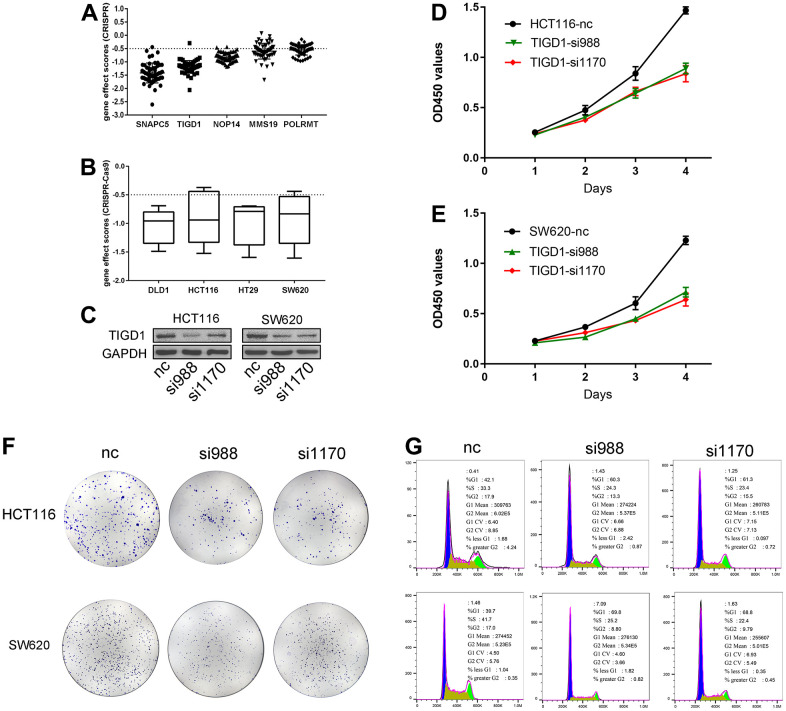
**Gene effect scores based on CRISPR-Cas9 and Silencing TIGD1 gene inhibited CC cells proliferation and cycle.** (**A**) The interference values of five CDM risk genes. (**B**) The interference values of colon cancer cell lines (DLD1, HCT116, HT29, SW620). (**C**) siRNAs interfered TIGD1 expression in HCT116 and SW620 (three group: nc, si988 and si1170). (**D**, **E**) HCT116 and SW620 cells were significantly inhibited in growth with knocking down the *TIGD1* gene expression by cck8 assays. (**D**) for HCT116, (**E**) for SW620. (**F**) The cloning ability of HCT116 and SW620 cell lines were significantly inhibited with knocking down the *TIGD1* gene expression by clone formation assays. (**G**) Silencing the expression of *TIGD1* gene retarded the proliferation cycle of HCT116 and SW620 in the G1 phase.

CRISPR-Cas9 is a relatively mature gene editing technology that is widely used in basic and clinical settings. In this study, the interference effects of genes associated with the CDM signature were deeply mined on the CDM platform by using CRISPR-Cas9 technology. As Figure 10A shows, the interference values of all five risk genes were less than -0.5, with the best interference value associated with the knockout SNAPC5 gene and the worst being with POLRMT, both of which are important factors affecting CC cell growth. After further statistical analysis, it was found that the growth of two types of CC cells, DLD1 and HCT116, was most affected by this model, followed by SW620, and finally HT29 (Figure 10B).

To determine whether the *TIGD1* genes is hub genes in CC, we designed siRNAs for the interference sites of *TIGD1*. As shown in Figure 10C, siRNAs interfering with *TIGD1* had a good inhibitory effect in SW620 and HCT116 cells. Cell proliferation was detected by clone formation and CCK8 assays. The growth of both HCT116 and SW620 cells was significantly inhibited by knocking down the *TIGD1* (Figure 10D, 10E). In addition, the cloning ability of the cells was also clearly affected by silencing the gene as shown in Figure 10F. The results of cell cycle experiments revealed that silencing the gene retarded the proliferation cycle of HCT116 and SW620 cells more or less in the G1 phase, thus retarding the cell growth rate (Figure 10G). This result is also consistent with our previous KEGG findings regarding the impact of CDM-related genes on the cell cycle (Supplementary Figure 1B).

### WGCNA and CDM immune subtype construction

**Figure 11:**
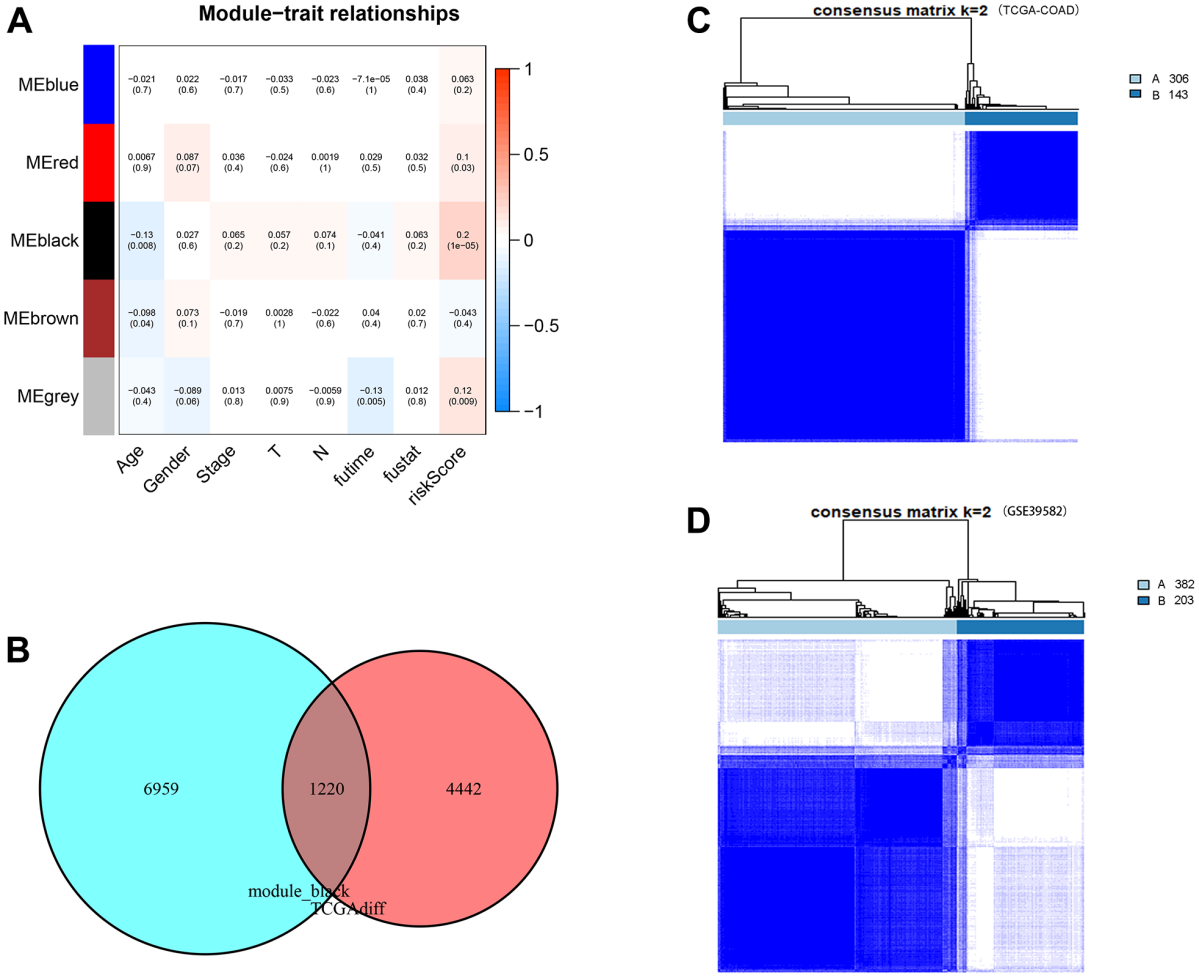
**WGCNA analysis, consensus clustering and immune subtype construction.** (**A**) Module–Clinical Trait Relationships of consensus module eigengene and different clinical features of COAD. Each row in the table corresponds to a module eigengene (ME), and each column to a clinical parameter (five module: blue, red, black and grey; eight clinical features: Age, Gender, Stage, T, N, futime, fustat and CDM riskScore). Each cell contained the correlation coefficients and p value (red, positively correlated; green, negative correlated). (**B**) Venn plot of CDM-associated co-expressed genes (5,662 genes) and upregulated genes (8,179 genes). Red represents coexpressed genes and cyan represents TCGA-COAD upregulated genes. (**C**) The consensus score matrix of TCGA-COAD samples when k = 2. Subtype A contains 306 samples and subtype B contains 143 samples. (**D**) The consensus score matrix of GSE39582 samples when k = 2. Subtype A contains 382 samples and subtype B contains 203 samples.

WGCNA was used to analyse coexpressed gene clusters associated with CDM signature scores based on the expression profiles in the TCGA-COAD data. The power value was chosen to be three to ensure a scale-free network (R^2^=0.9, Supplementary Figure 7A, 7B). Five coexpression models were clustered in the hierarchical clustering tree (Supplementary Figure 7C), with the black module having the strongest positive correlation with the CDM risk scores (Cor=0.2, P = 4e^-05^, Figure 11A). The black module contained 5,662 coexpressed genes, there were 8,179 TCGA-COAD differentially upregulated genes, and the two together included 1,220 differentially coexpressed upregulated genes (Figure 11B).

To define new immune subtypes of CC based on the coexpressed upregulated genes in the CDM signature, the TCGA-COAD and GSE39582 samples were clustered using the consensus clustering method [34]. The optimal k value was determined using cophenetic correlation coefficients, and the CDF curve had the flattest middle segment when k = 2 (Supplementary Figure 7D, 7E). In addition, the consensus matrix heatmap also maintained clear boundaries at k = 2. Therefore, the optimal number of subtypes was determined to be k = 2, and two new immune subtypes named “subtype A” (n = 306) and “subtype B” (n = 143) in TCGA-COAD samples, and “subtype A” (n = 382) and “subtype B” (n = 203) in GSE39582samples were identified (Figure 11C, 11D). Subsequently, the heatmap of Supplementary Figure 7F shows the expression of 1,220 coexpressed upregulated genes in immune subtype A and B TCGA-COAD samples. It is clear that the CDM-associated gene expression in subtype A is significantly higher than that in subtype B samples.

### New immune subtypes A and B distinguish immune benefit patients

**Figure 12:**
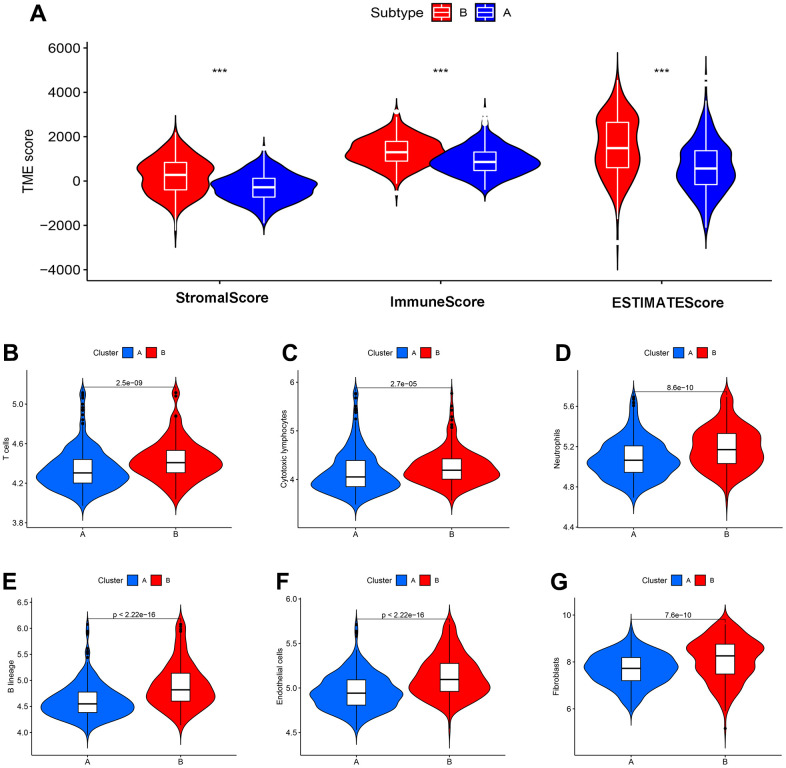
**CDM subtypes associated with immunotherapeutic response predictors in GSE39582 datasets.** (**A**) Tumor immune microenvironment analysis between CDM immune subtype A and subtype B by Microenvironment Cell Populations counter algorithm. (**B**–**G**) The association between CDM subtypes and immune cell infiltration; (**B**) for T cells; (**C**) for cytotoxic lymphocytes; (**D**) for neutrophils; (**E**) for B lineages; (**F**) for endothelial cells; (**G**) for fibroblasts.

**Figure 13:**
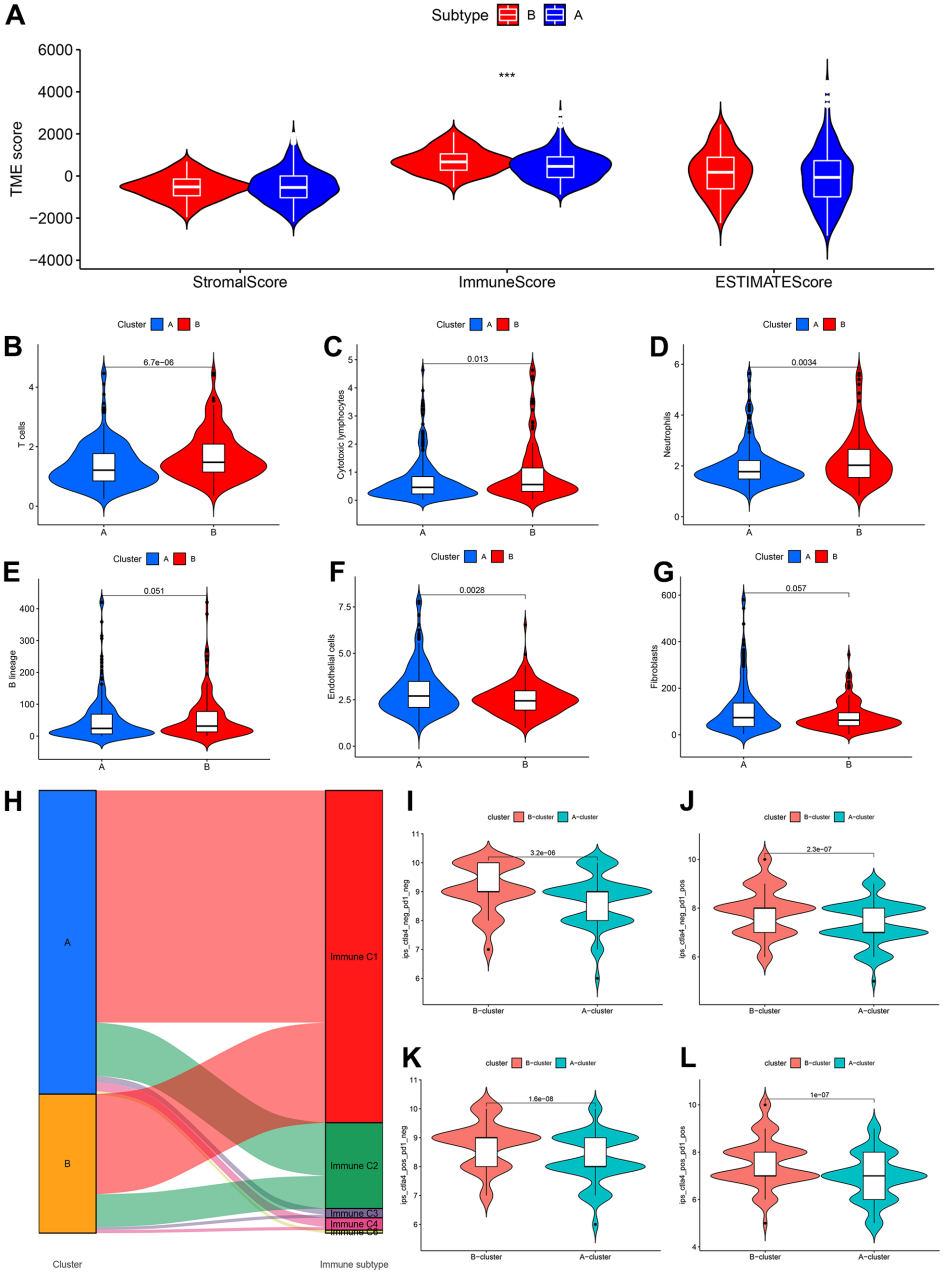
**CDM subtypes associated with immunotherapeutic response predictors in TCGA-COAD datasets.** (**A**) Tumor immune microenvironment analysis between CDM immune subtype A and subtype B by Microenvironment Cell Populations counter algorithm. (**B**–**G**) The association between CDM subtypes and immune cell infiltration; (**B**) for T cells; (**C**) for cytotoxic lymphocytes; (**D**) for neutrophils; (**E**) for B lineages; (**F**) for endothelial cells; (**G**) for fibroblasts. (**H**) Sankey diagram between the CDM subtypes and immune subtypes (C1-C6). (**I**–**L**) Relationship between the CDM subtypes and the immunotherapy data of PD1 and CTLA4 (**I**) for PD1 and CTLA4 negative expression; (**J**) for PD1 positive expression and CTLA4 negative expression; (**K**) for CTLA4 positive expression and PD1 negative expression; (**L**) for PD1 and CTLA4 negative expression.

Tumour immune microenvironment analysis revealed that the sample of subtype B exhibited stronger immune cell infiltration than subtype A, in particular GSE39582 cohort (Figure 12A). Furthermore, Figure 12B–12G were analyzed the abundance of immune cells from subtypes A and B in GSE39582 samples. In TCGA-COAD samples, tumour immune microenvironment analysis revealed a higher ImmuneScore in subtype B than in subtype A samples, while there was no difference in ImmuneStroma and ESTIMATEScore, indicating that there is a definite link between the two new CDM molecular subtypes and immune cell infiltration (Figure 13A). Based on this, we found that cells with tumour-killing properties, T cells, cytotoxic lymphocytes, neutrophils, and B cells were significantly more abundant in subtype B than in subtype A samples by the MCP-counter immune algorithm; however, the opposite result was found for immunosuppressive cells, endothelial cells and fibroblasts (Figure 13B–13G). According to the article “The Immune Landscape of Cancer” published in the journal Immunity in 2018, an immunogenicity analysis of 33 cancers in TCGA was performed, and all tumours were classified into six immune subtypes, namely, wound healing (Immune C1), IFN-dominant (Immune C2), inflammatory (Immune C3), lymphocyte depleted (Immune C4), immunologically quiet (Immune C5) and TGF-dominant (Immune C6) [35]. As shown in Figure 13H, a Sankey diagram illustrated the relationship between the CDM immune subtypes and the six immune subtypes. Further statistics showed that the proportion of the C2 population with immune benefit in the subtype B samples was significantly higher than that in the subtype A samples, which was consistent with the previous results (Supplementary Figure 7G–7H). With the above analysis of the immune correlation between the two subtypes, patients with subtype B may have a better prognostic benefit from an immunotherapy perspective. Using TCGA-COAD immunotherapy data from TCIA, we analysed the relationship between the two subtypes and the immunotherapy data of PD1 and CTLA4 in the TCGA-COAD samples. The subtype B patients had better treatment outcomes (Figure 13I–13L).

## DISCUSSION

The morbidity of CC ranks among the top three in the world representing a very high proportion of all malignant tumours, and posing a serious threat to human life, health, and property security. Although the treatment concept and technical means of CC have been greatly improved, poor patient prognosis management, the occurrence of multidrug resistance and the high recurrence rate of the tumour remain major issues. Personalized patient management and optional multilocus targeted therapy can minimize tumour recurrence and prolong survival time, creating a promising prognosis for patient survival. CRISPR-Cas9 technology, a cornerstone of research for screening functional genes, is an effective method for discovering tumour-dependent genes. CDMs have been developed to detect tens of thousands of tumour-dependent genes in thousands of tumour cell lines via using CRISPR-Cas9 and are being updated continuously, thus offering an abundant resource and a solid foundation for this study. Based on the basic framework of CDM, Kenichi et al. developed a ShinyDepMap web server (https://labsyspharm.shinyapps.io/depmap) [36] that predicts and evaluates the efficacy of gene-specific drugs, while also retrieving the most sensitive cancer cell lines for the specified drugs. Moreover, Chiu and his colleagues [37] introduced the use of deep learning in CDM, which more quickly and more effectively predicts cancer dependencies. Apart from that, Neekesh V [38] established a first-generation paediatric CDM, that included 13 different forms of paediatric solid and brain tumours.

First, CC dependent gene screening results from the CDM combined with TCGA-COAD differentially expressed genes identified 1,304 overexpressed and CC-dependent oncogenes. Then, the CDM signature with five cancer-dependent genes and prognostic nomogram were constructed by Lasso Cox regression and multivariate Cox analyses, and they demonstrated robust predictive performance and close relationship with clinical characteristics in different datasets. Furthermore, WGCNA and consensus clustering were used to define coexpressed genes with the CDM risk scores and identify two new immune subtypes. Finally, we conducted a comprehensive investigation of the relationship between the two subtypes and immune regulation and the immune microenvironment and the impact of immunotherapy based on the different subtypes.

The CDM signature comprised the *MMS19, NOP14, POLRMT, SNAPC5* and *TIGD1* genes. Statistical analysis revealed that these genes were highly expressed in CC tissues and were tumour-dependent genes from CDMs. Experiments revealed that the TIGD1 genes were oncogenes that impacted the CC cell cycle. Li et al. also [16] discovered that *TIGD1*, an unidentified gene, is substantially expressed in colorectal, gastric, liver, lung, and pancreatic cancers; may be involved in tumour cell cycle regulation; and is negatively correlated with prognosis. Zhu [14] discovered that NOP14 regulates the NRIP1/GSK-3β/β-catenin signalling pathway to promote colorectal cancer proliferation and migration. In pancreatic cancer [39], NOP14 is highly expressed and promotes tumour invasion and metastasis by targeting p53 mutation. In contrast, some studies have reported that NOP14 inhibits melanoma by regulating the Wnt/β-catenin pathway [40] and cancer stemness [41] and inhibits breast cancer by inhibiting the NRIP1/Wnt/β-catenin pathway [42]. In combination with Zhu’s study, we found that *NOP14* mRNA is abundantly expressed in CC tissues; however, it is a protective prognostic factor for CC patients, which seems to be a contradiction. Of course, it is unfortunate that the relationship between the expression of NOP14 and CC patient prognosis was not explored in Zhu’s study. The existing research on NOP14 in CC is just the tip of the iceberg, and the specific mechanism deserves to be further explored.

We further explored the relationship between the CDM signature and the IC50 value of targeted drugs when elucidating the association between this signature and the prognostic role and clinical characteristics of CC. These targeted drugs are mainly composed of tyrosine kinase inhibitors (AMG.706: motesanib, AP.24534: ponatinib, axitinib, AZD0530, AZD7762, imatinib and nilotinib), a p21-activated kinase inhibitor (IPA3), and a traditional Chinese medicine (shikonin). Drug sensitivity analysis found that patients with low CDM risk scores were sensitive to tyrosine kinase inhibitors. In addition, the previous KEGG and GO analyses revealed that the highly expressed CDM-related genes were mainly involved in the cell proliferation cycle and regulated the proliferation of CC cells. Ho et al. [43] found that AMG.706 exerts an anticancer effect by regulating cell cycle progression and apoptotic cell death. IPA3, a small-molecule p21-activated kinase inhibitor, has excellent antitumour effects by enforcing cell cycle arrest and inducing apoptosis [44, 45]. Shikonin, a naphthoquinone compound extracted from Lithospermum, has been extensively studied for its antitumour activity. In CC, shikonin can arrest the cell cycle by inhibiting the hypoxia-inducible factor signalling pathway [46], promote cell apoptosis by downregulating the PI3K/Akt signalling pathway [47], and inhibit growth by regulating the STAT3 signalling pathway [48]. Therefore, we hypothesized that the tumour cells of patients with high CDM risk have self-regulated cell cycle activity to resist the tumour-killing effects of these drugs, especially tyrosine kinase inhibitors.

As Volker reported, vioprolide A, a natural product, has an outstanding therapeutic effect for acute lymphoblastic leukaemia by targeting NOP14 expression to affect ribosome biogenesis [49]. The results of the KEGG pathway and GO functional analyses are consistent with the conclusions drawn from Volker’s study. *POLRMT*, a core mitochondrial gene, is responsible for mitochondrial metabolism, and inhibition of POLRMT expression can be utilized to induce an energy crisis from multidrug resistant cells and thus resensitize them to chemotherapeutic agents [50]. In a tumour cell reconstituted recombinant system, the inhibitor of mitochondrial transcription (IMTs), which specifically targets *POLRMT*, significantly hampers mitochondrial DNA (mtDNA) transcription and triggers the dose-dependent inhibition of mtDNA expression and the oxidative phosphorylation system [17]. According to Eliseo’s report [51] based on supervised support vector machine learning, the high expression of TIGD1, to a certain extent, affects the sensitivity of tumour cells to cisplatin and improves patient outcomes after chemotherapy. The drugs shown in Figure 6 have good clinical potential for patients at high risk according to the CDM signature, especially patients with advanced multidrug resistant disease, according to the CDM signature for drug resistance analysis. A recent study found that knockdown of TIGD1 was effective in accelerating copper death in colorectal cancer cells induced by the copper death inducer elesclomol, and thus TIGD1 could serve as a key protein in regulating copper-dependent cell death [52]. Qiao et al. [53] found that TIGD1, which was significantly highly expressed in oral squamous cell carcinoma, effectively promoted the proliferation and invasive metastasis of cancer cells and was also involved in immune cell infiltration in tumor tissues, thus promoting the progression of oral squamous cell carcinoma.

Based on the CDM-related coexpression of differential genes, the consensus clustering divided the TCGA-COAD and GSE39582 samples into two new subtypes A and B. In the overall analysis, immune cell scores were significantly higher in the B molecular subtype than the A subtype, and tumour-killing immune cells, such as T cells, B cells, cytotoxic lymphocytes, and neutrophils, were also highly infiltrated in the B molecular subtype samples. From another perspective, all samples with Immune C6 were subtype A. In addition, in terms of PD1 and CTLA4 immune therapy benefit, subtype A did not perform as well as subtype B. These results also reflect the fact that subtype A is associated with a poorer immune benefit than the subtype B.

Although this study is founded on a significant amount of TCGA-COAD, GSE17536 and GSE39582 data, there are still some limitations. The present study is based on a retrospective analysis of a public database of CC samples, which lacks prospective clinical application and validation. Thus, more research is necessary to comprehensively guide CC clinical diagnosis and treatment.

## Supplementary Material

Supplementary Figures

Supplementary Table 1
