# Supplementary material for: Exploring Cancer Dependency Map genes and immune subtypes in colon cancer, in which TIGD1 contributes to colon cancer progression
Source: Aging (Albany NY). 2023 Jul 13;15(13):6400–28. doi: 10.18632/aging.204859 (PMC10373975; doi:10.18632/aging.204859)
Supplement: Supplementary Figures [file aging-15-204859-s001.pdf]

SUPPLEMENTARY FIGURES

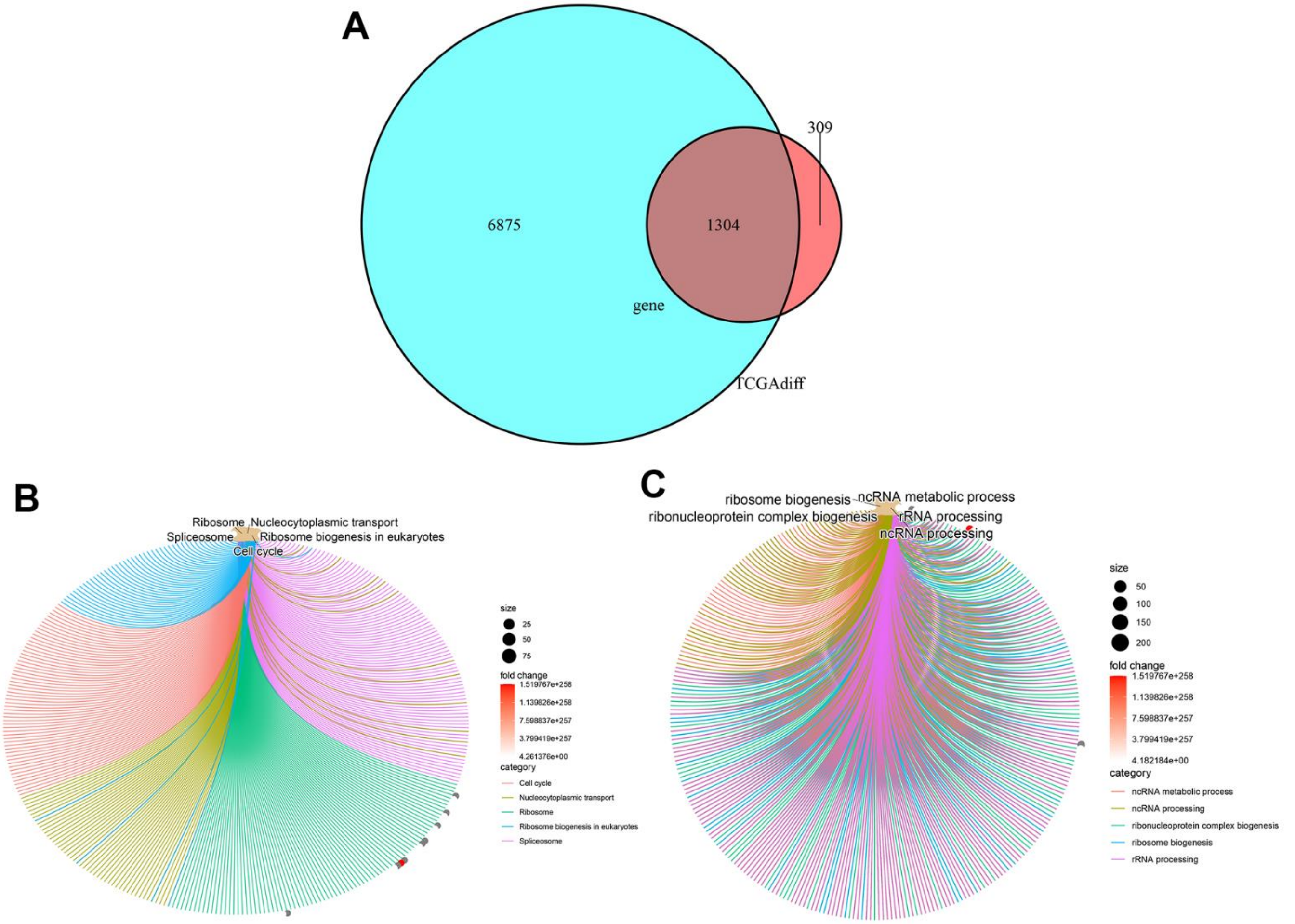

Supplementary Figure 1. Venn and circle graph of KEGG and GO in differentially expressed CDM genes. (**A**) Venn plot. 1,304 differentially expressed CDM genes between differentially expressed genes of TCGA-COAD and CDM genes. Cyan shows 8,179 differentially expressed genes in TCGA-COAD, dull red shows 1,613 cancer-dependent CDM-associated genes and overlapping area shows the 1,304 differentially expressed CDM genes. (**B**) Circle plot. KEGG pathway enrichment of 1304 differential expression of CDM genes. (**C**) Circle plot. GO Biological process analysis of 1304 differential expression of CDM genes.

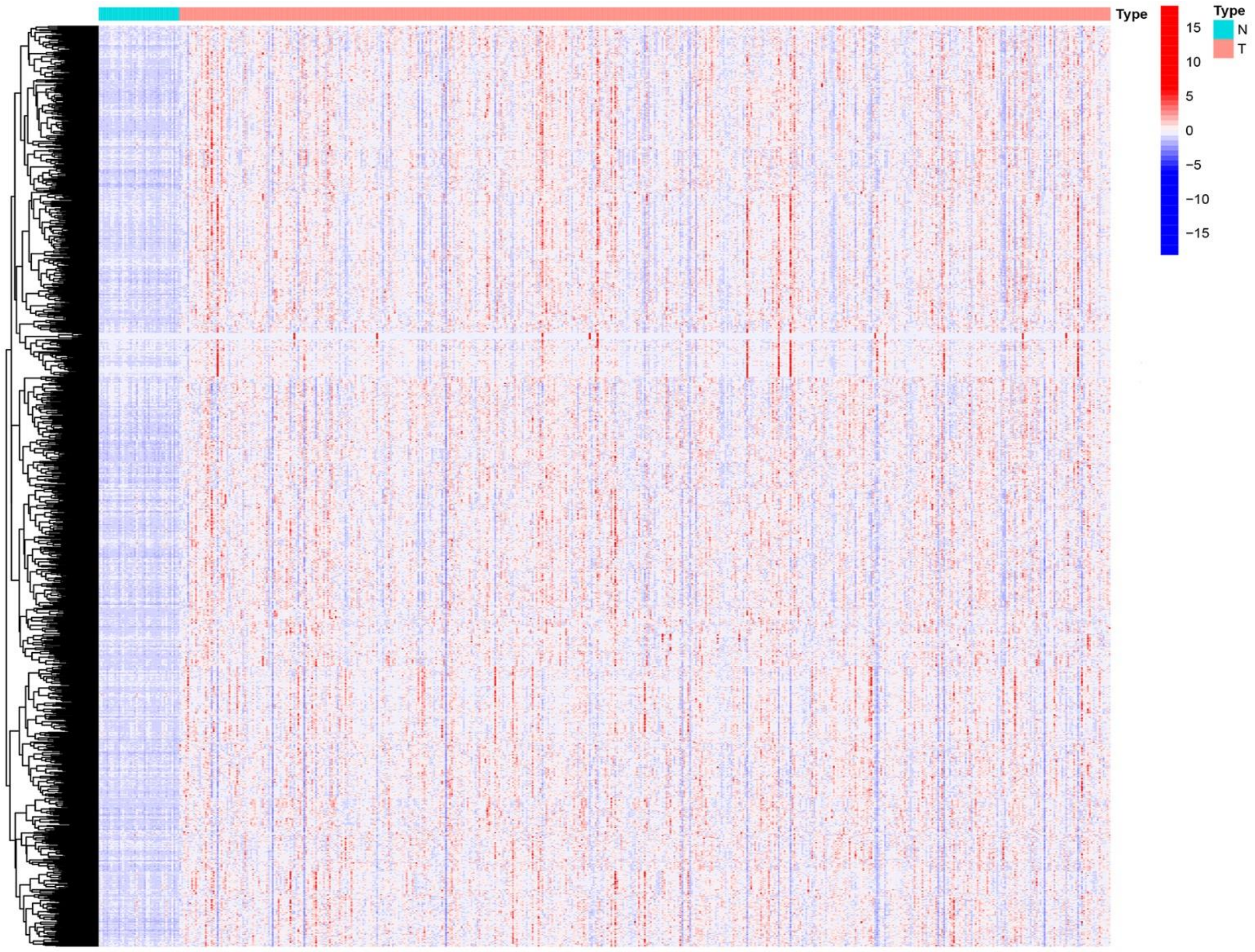

Supplementary Figure 2. Heatmap 1,304 differentially expressed CDM genes between differentially expressed genes of TCGA-COAD.

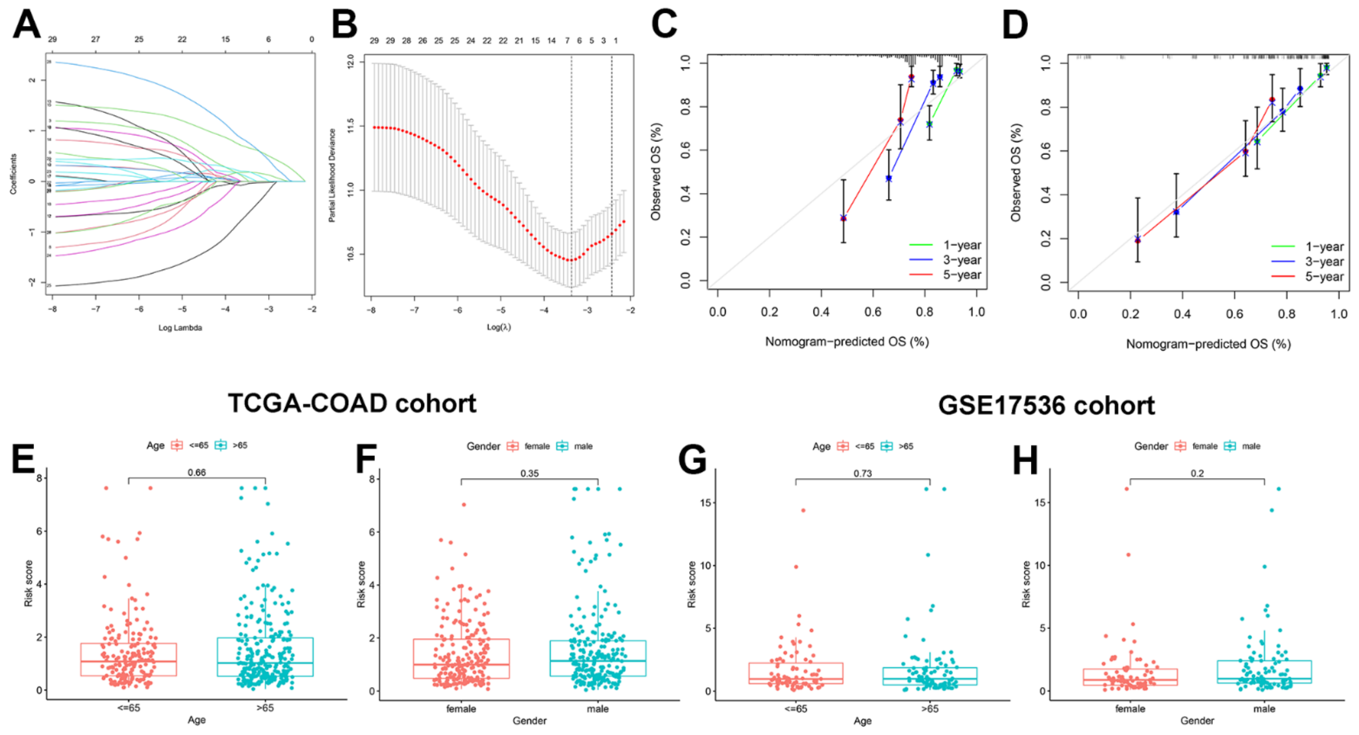

Supplementary Figure 3. Least absolute shrinkage and selection operator (LASSO) Cox regression analysis and nomogram calibration. (**A**, **B**) LASSO Cox regression analysis in TCGA-COAD cohort. (**A**) LASSO coefficient profiles of the 29 prognosis-correlated CDM genes. (**B**) Genes from univariate Cox regression analysis were narrowed down by the lasso algorithm, The left vertical line represents the 5 CDM genes finally identified. (**C**, **D**) The calibration plot of the CDM nomogram for predicting 1-year(green), 3-year(blue) and 5-year(red) survival rates; (**C**) for TCGA-COAD cohort, (**D**) for GSE17536. (**E**) Boxplot. Correlation between age and risk-CDM score in TCGA-COAD cohort. Pink represents age less than or equal to 65 years old and cyan represents age greater than 65 years old (p=0.66). (**F**) Boxplot. Correlation between gender and risk-CDM score in TCGA-COAD cohort. Pink represents female and cyan represents male (p=0.35). (**G**) Boxplot. Correlation between age and risk-CDM score in GSE17536 cohort. Pink represents age less than or equal to 65 years old and cyan represents age greater than 65 years old (p=0.73). (**H**) Boxplot. Correlation between gender and risk-CDM score in GSE17536 cohort. Pink represents female and cyan represents male (p=0.2).

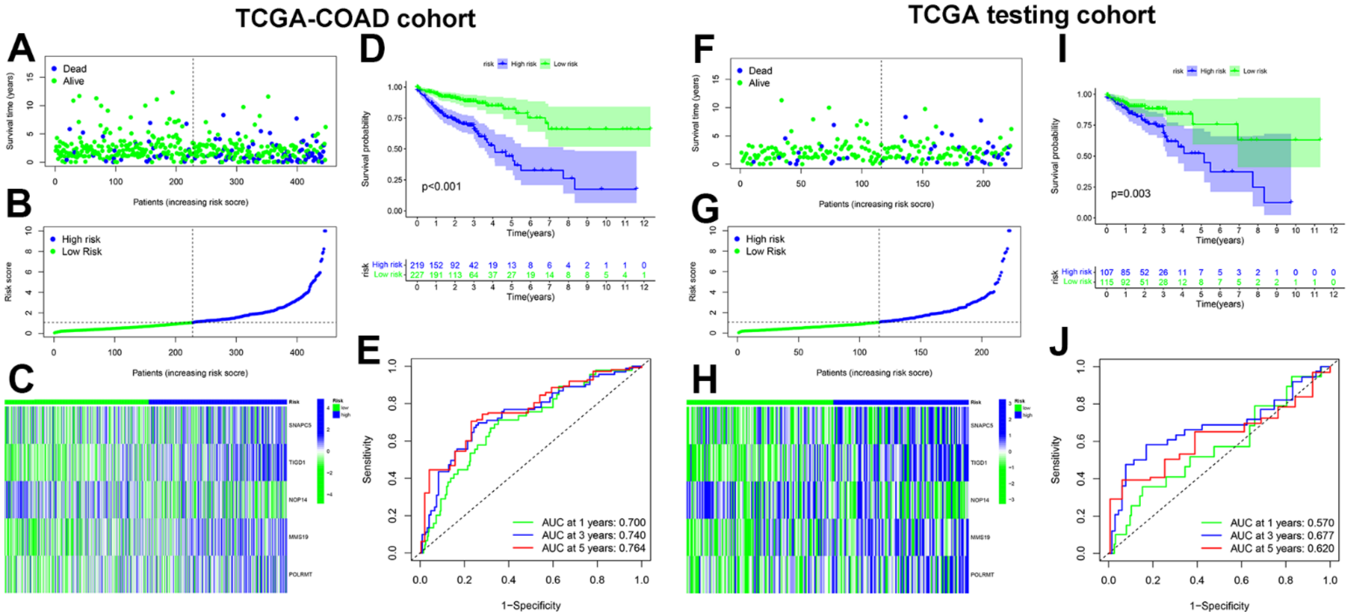

Supplementary Figure 4. Cancer Dependency Maps (CDMs) signature construction and validation in all TCGA-COAD cohort and TCGA testing cohort. (**A**) Survival scatter plot in all TCGA-COAD cohort. A dot represent a CC patient (blue represent Dead, Green represent alive). Dotted lines show the median of risk score that dichotomize patients into high and low groups. (**B**) Risk score plot in all TCGA-COAD cohort. The CC patients are ordered by the risk score of the CDM signature. Dotted lines show the median of risk score that dichotomize patients into high (blue) and low (green) groups. (**C**) Heatmap of five CDM signature genes (*SNAPC5*, *TIGD1*, *NOP14*, *MMS19* and *POLRMT*) in all TCGA-COAD cohort. Blue represent high-CDM risk group and green represent low-CDM risk group. (**D**) Kaplan-Meier plot in all TCGA –COAD cohort (p<0.001). Survival curves for high (blue) and low (green) risk-CDM groups dichotomized at the median of risk-CDM score are plotted. 95% confidence intervals for each group are also indicated by shadow area. (**E**) The ROC curve of CDM signature in all TCGA-COAD cohort. Green show one year AUC value (AUC value=0.700), blue show three year AUC value (AUC value=0.740) and red show five year AUC value (AUC value=0.764). (**F**) Survival scatter plot in TCGA testing cohort. (**G**) Risk score plot in TCGA testing cohort. (**H**) Heatmap of five CDM signature genes (*SNAPC5*, *TIGD1*, *NOP14*, *MMS19* and *POLRMT*) in TCGA testing cohort. (**I**) Kaplan-Meier plot in TCGA testing cohort (*p*=0.003). (**J**) The ROC curve of CDM signature in TCGA testing cohort. Green show one year AUC value (AUC value=0.570), blue show three year AUC value (AUC value=0.670) and red show five year AUC value (AUC value=0.620).

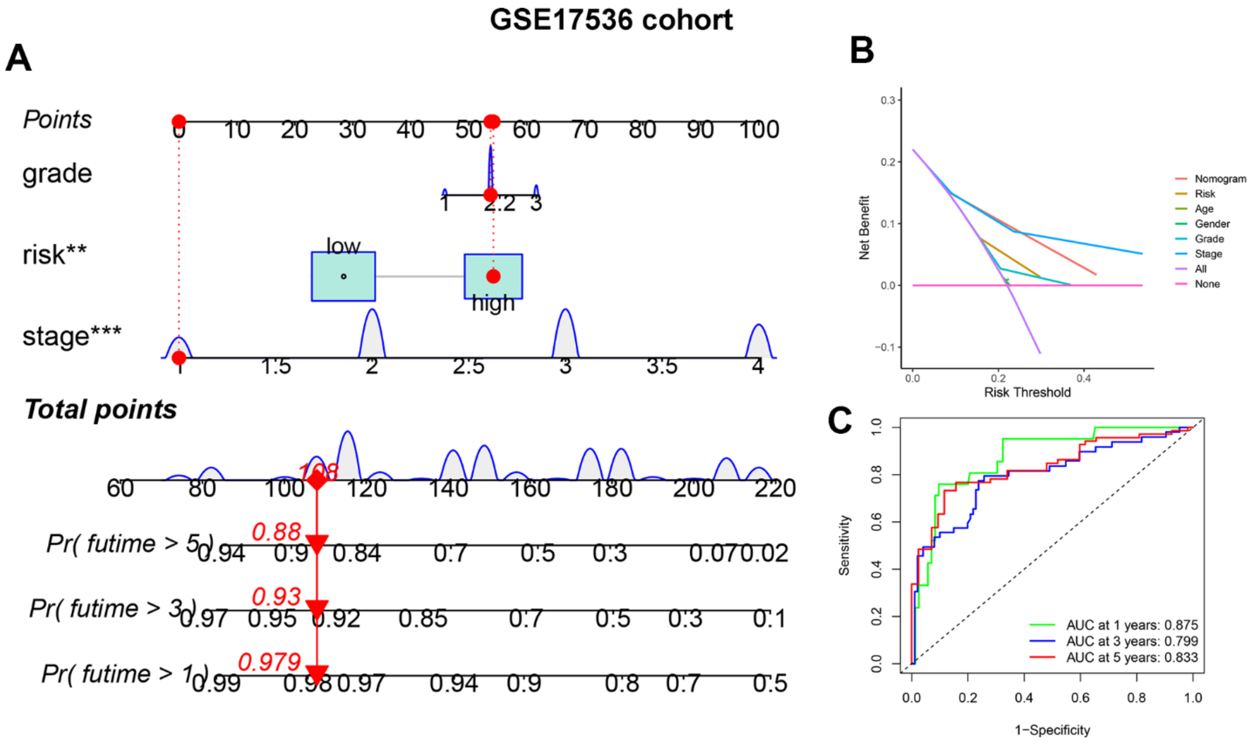

Supplementary Figure 5. CDM-associated nomogram validation in GSE17536 cohort. (**A**) CDM-associated Nomogram for predicting 1-, 3-, and 5-year OS of patients in GSE17536 cohort. (**B**) DCA analysis for CDM-associated nomogram in GSE17536 cohort. (**C**) The ROC curve of CDM-associated Nomogram in GSE17536 cohort. Green show one year AUC value (AUC value=0.875), blue show three year AUC value (AUC value=0.799) and red show five year AUC value (AUC value=0.833).

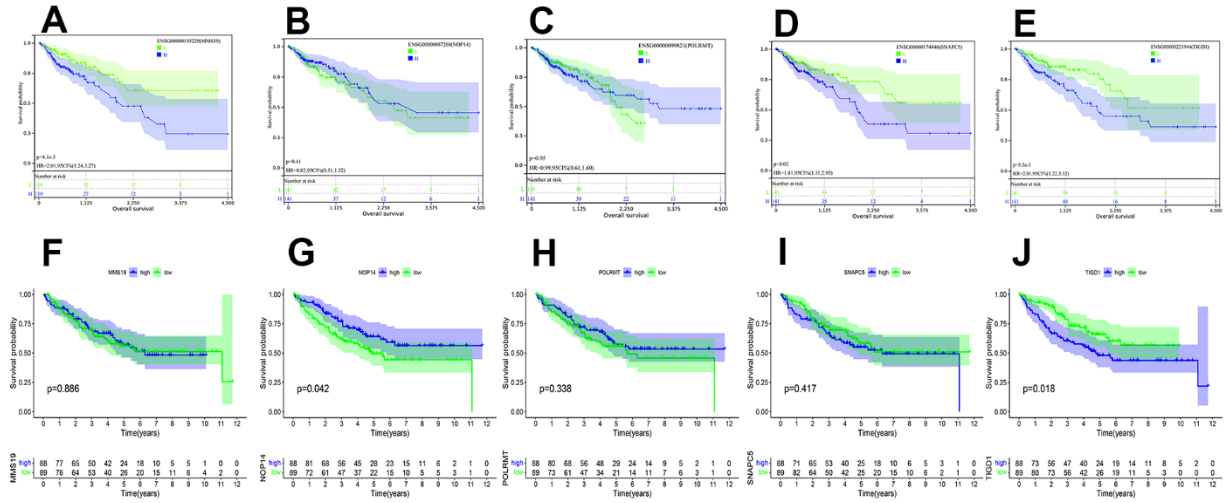

Supplementary Figure 6. Kaplan-Meier plot for five CDM signature genes (MMS19, NOP14, POLRMT, SNAPC5 and TIGD1) in all TCGA–COAD and GSE17536 cohort (p<0.001). Survival curves for high (blue) and low (green) expression groups dichotomized at the median expression are plotted. 95% confidence intervals for each group are also indicated by shadow area. (**A**) MMS19 in TCGA–COAD (p=4.1e3). (**B**) NOP14 in TCGA–COAD (p=0.41). (**C**) POLRMT in TCGA–COAD (p=0.95). (**D**) SNAPC5 in TCGA–COAD (p=0.02). (**E**) TIGD1 in TCGA–COAD (p=5.5e3). (**F**) MMS19 in GSE17536 cohort (p=0.886). (**G**) NOP14 in GSE17536 cohort (p=0.042). (**H**) POLRMT in GSE17536 cohort (p=0.338). (**I**) SNAPC5 in GSE17536 cohort (p=0.417). (**J**) TIGD1 in GSE17536 cohort (p=0.018).

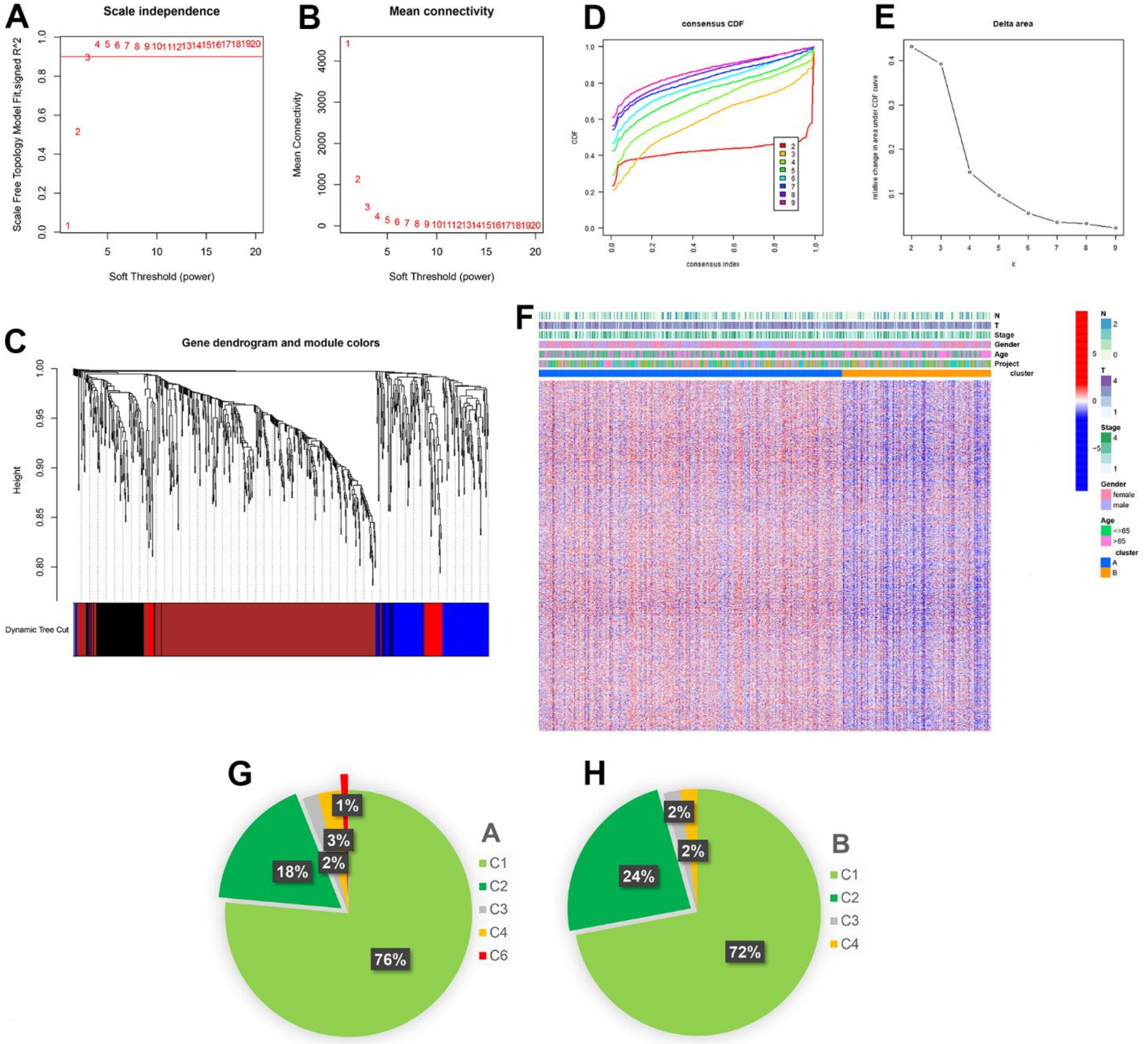

Supplementary Figure 7. WGCNA analysis, consensus clustering and heatmap of two immune subtypes. (**A**, **B**) The scale-free fit index for soft-thresholding powers. (**A**) The relationship between the soft-threshold and scale-free R2. (**B**) The relationship between the soft-threshold and mean connectivity. (**C**) Different modules are labeled in different colors and dendrogram of all genes clustered in TCGA-COAD. (**D**, **E**) Identification of CDM molecular subtypes; (**D**) The cumulative distribution function curves of consensus scores based on different subtype numbers (k = 2~9); (**E**) The comparison of the relative changes of the area under the CDF curve between k and k–1. (**F**) The heatmap of 1220 differential expression of CDM co-expressed genes in TCGA-COAD Subtype A and Subtype B. (**G**, **H**) The proportion of C1-C6 six immune subtypes in subtype A and subtype B; (**G**) for subtype A; (**H**) for subtype B.
