# Supplementary material for: Exploring Cancer Dependency Map genes and immune subtypes in colon cancer, in which TIGD1 contributes to colon cancer progression
Source: Aging (Albany NY). 2023 Jul 13;15(13):6400–28. doi: 10.18632/aging.204859 (PMC10373975; doi:10.18632/aging.204859)
Supplement: Supplementary Table 1 [file aging-15-204859-s002.pdf]

SUPPLEMENTARY TABLE

Supplementary Table 1. 29 prognostic related genes were discovered by univariate Cox hazard analysis in COAD.

id	HR	HR.95L	HR.95H	pvalue
PSMA5	0.589785	0.372309	0.934294	0.024479
SF3A2	1.555346	1.004943	2.407203	0.047469
USP5	1.724162	1.004683	2.958878	0.048051
POLR2H	1.908472	1.137331	3.202469	0.014396
SNAPC4	1.809706	1.171914	2.794604	0.007461
ELL	1.855009	1.063099	3.236817	0.029601
PSMD12	0.640136	0.41484	0.987789	0.043855
SUPT5H	1.73605	1.033636	2.915795	0.037068
EIF2B5	2.217337	1.131043	4.346947	0.020423
BRF1	1.851907	1.008635	3.400201	0.046845
SNAPC5	1.773781	1.111222	2.831388	0.016308
CDK9	1.944925	1.150271	3.288557	0.013051
SART1	1.661597	1.052816	2.622399	0.029181
PNN	1.463655	1.000863	2.14044	0.049483
TIGD1	1.679921	1.228964	2.296353	0.001143
GTF2E2	0.671251	0.473699	0.95119	0.025004
TAF1C	1.866283	1.237083	2.815504	0.002939
INTS1	1.467568	1.019766	2.112012	0.03889
ERCC3	1.992833	1.071324	3.706989	0.029441
CIAO1	2.052983	1.029583	4.093638	0.041076
PELP1	1.499056	1.030459	2.180745	0.034272
SNAPC3	1.629411	1.053366	2.520475	0.028267
DDX11	1.637622	1.121073	2.392178	0.01074
RAD9A	1.596121	1.015991	2.507504	0.042479
NOP14	0.619611	0.401996	0.955029	0.030127
DHX38	1.60973	1.003591	2.581959	0.048287
SFSWAP	1.931741	1.078866	3.458841	0.026734
MMS19	2.041935	1.171788	3.558236	0.011754
POLRMT	1.546128	1.034675	2.3104	0.033479
